# The Kink-Turn 7 Motif: An Additional Test for RNA Force Field Performance

**DOI:** 10.1021/acs.jctc.5c00776

**Published:** 2025-12-08

**Authors:** Toon Lemmens, Vojtěch Mlýnský, Jiří Šponer, Martin Pykal, Pavel Banáš, Michal Otyepka, Miroslav Krepl

**Affiliations:** 1 Institute of Biophysics of the Czech Academy of Sciences, Královopolská 135, Brno 612 00, Czech Republic; 2 National Centre for Biomolecular Research, Faculty of Science, Masaryk University, Kamenice 5, Brno 625 00, Czech Republic; 3 Czech Advanced Technology and Research Institute, CATRIN, Palacký University, Křížkovského 511/8, Olomouc 779 00, Czech Republic; 4 IT4Innovations, VSB-Technical University of Ostrava, 17. listopadu 2172/15, Ostrava-Poruba 708 00, Czech Republic

## Abstract

The kink-turn is a recurrent RNA structural motif that induces a sharp bend (kink) in the A-form RNA helix. It is defined by key structural features, including consecutive sheared AG base pairs, an A-minor interaction, and multiple base–sugar interactions. An accurate representation of these densely packed noncanonical interactions by molecular dynamics simulations poses a significant challenge for contemporary force fields (FFs). Here, we present extended simulations of the ribosomal kink-turn 7 (Kt-7) from *H.m.*, the so-called “consensual” kink-turn, using a broad spectrum of pair-additive and polarizable RNA FFs. None of the tested FFs manage to flawlessly describe all of the structural features of the Kt-7 although several FFs provide rather acceptable results and should not cause problems in simulations of larger RNAs containing a kink-turn. On aggregate, the widely used OL3 (ff99bsc0χ_OL3_) and polarizable AMOEBA FFs achieve the best performance for this motif. Interestingly, some more recently parametrized FF variants struggle to describe the Kt-7’s tertiary A-minor interaction – a ubiquitous tertiary contact in RNA. This raises some concerns about the broader applicability of these FFs and suggests that they may be overfitted to small model systems, such as RNA tetranucleotides. In some cases, irreversible unkinking of the entire kink-turn motif can also be observed. The kink-turn motif is highly sensitive to variations in RNA FFs, and we strongly recommend its inclusion in training and benchmarking data sets as an important regression test to improve the robustness and accuracy of RNA FF parametrization.

## Graphical abstract

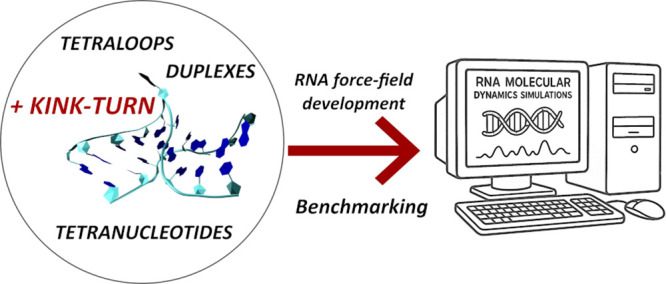

## Introduction

The kink-turn is a recurrent structural motif, widespread in RNA across all domains of life,^1,2^ where it contributes to a myriad of structural and biological functions. Kink-turns mediate protein–RNA and RNA–RNA tertiary contacts in ribosomes,^3^ form crucial structural elements in several riboswitches,^4–6^ and are involved in the assembly of the eukaryotic spliceosome.^7,8^ They are a powerful tool in RNA nanotechnology, allowing for precise control over RNA folding and modular assembly in RNA origami and nanostructures.^9^ While their characteristic bent shape is strongly conserved, the individual kink-turns display sequence variations, especially in the bulge region.^10^

Kink-turn 7 (Kt-7) from the large ribosomal subunit of *Haloarcula marismortui* is considered as the consensual kink-turn, as it closely matches the average sequence of known kink-turns while also exhibiting all the defining structural features of kink-turns (Figure 1).^1^ In other words, the structural properties of Kt-7 are highly representative of the general kink-turn motif, and it is the most widely studied kink-turn.^11–14^ Multiple highly similar X-ray structures of isolated Kt-7 are available in the database (Supporting Information Table S1).^15–17^ In all of them, Kt-7 is formed by a longer (b) and shorter (n) RNA strand, with the longer strand containing the three-nucleotide GAA bulge (loop). The structure further includes the canonical (C) and noncanonical (NC) stems separated by the bulge and containing the characteristic cis-Watson–Crick (*c*WW, canonical) GC and trans-Hoogsteen-sugar edge (*t*HS) AG base pairs, respectively.^18^ In Kt-7, as in every kink-turn motif, the characteristic bend (kink) is stabilized by a critical hydrogen bond (H-bond) between the first bulge nucleotide and the NC stem, along with an A-minor tertiary interaction bridging the C and NC stems (Figure 1).^3^ The first of these essential interactions is known as the kink-turn’s signature interaction (SI). It is formed between atoms G_L1_(O2’) and A_1n_(N1) and is utterly indispensable for the folding of all kink-turns (Figure 1B).^3^ Meanwhile, the A-minor interaction is formed between the A_2b_ nucleotide and the G_–1n_:C_–1b_ base pair.^3^ The A-minor interaction, where adenine is inserted into the minor groove of a canonical A-RNA duplex, is not only a critical stabilizing element for kink-turn motifs but also the most widespread tertiary interaction in RNA.^19,20^ The A-minor interaction can adopt four distinct substates (A-minor type 0, I, II, and III) that differ by their spatial orientation and H-bonding patterns.^21^ Only types 0 and I A-minor interactions are observed in kink-turns (Figure 1C), with the preference for a specific type determined by the nucleotide sequence as well as the kink-turn’s local structural context.^1,2^ For *H.m.* Kt-7, the type I is experimentally observed when the kink-turn is embedded in its ribosomal context while the type 0 is observed for isolated Kt-7 or when complexed with the L7Ae protein.^15^ There is no known X-ray crystallographic structure of a folded kink-turn, in which the SI and A-minor interactions are not formed.

**Figure 1:**
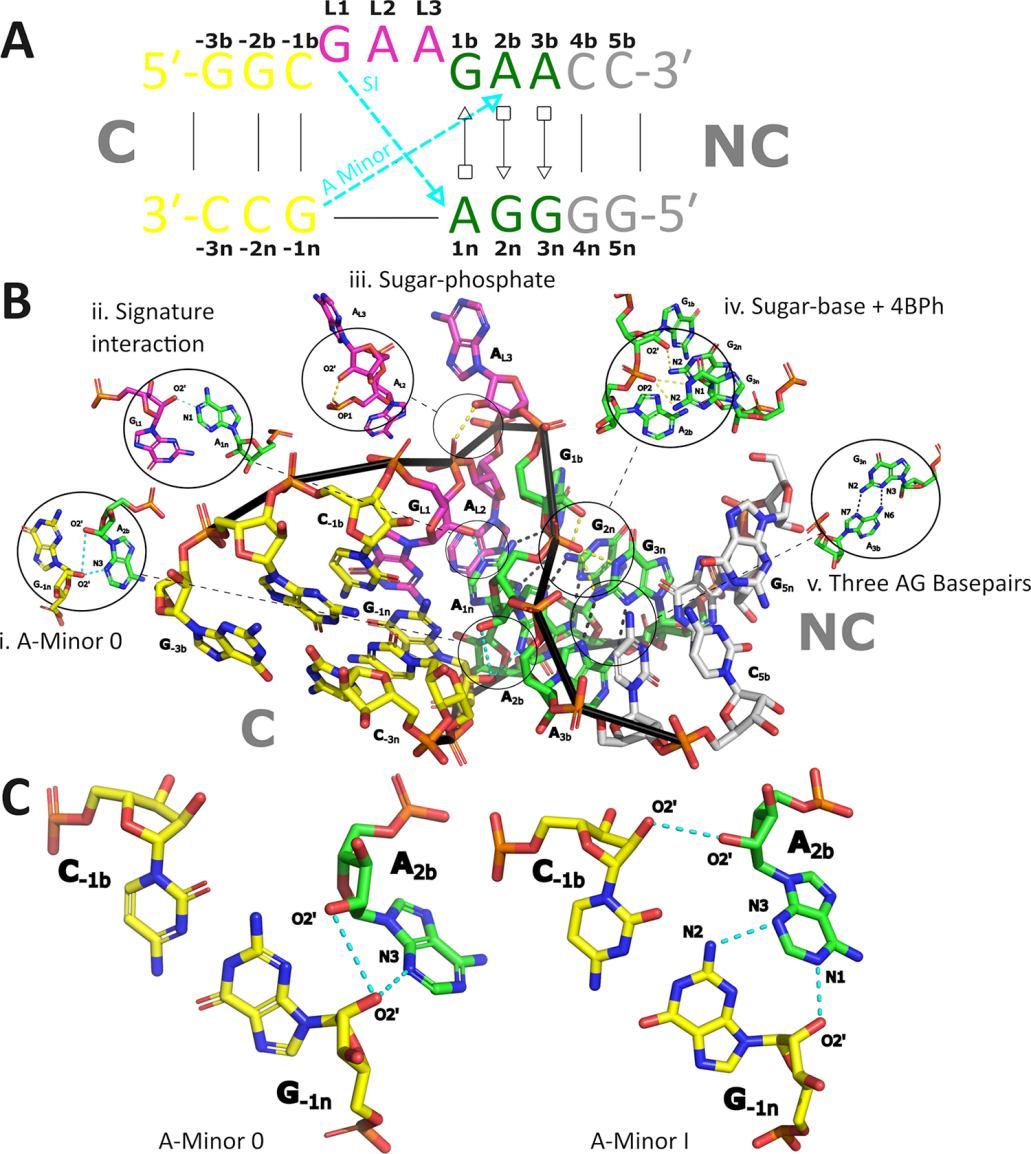
Structural overview of Kink-turn 7 (Kt-7) from *Haloarcula marismortui* (*H.m.*). (A) Schematic representation of Kt-7 using the Leontis & Westhof base pair classification.^18^ The Kt-7 from *H.m.* closely matches the consensual kink-turn sequence, making it an excellent representative of the motif.^1^ The canonical stem (C), the AG base pairs, the Watson–Crick base pairs of the noncanonical stem (NC), and the bulge are in yellow, green, gray and purple, respectively. The same color coding is used throughout this work. The main tertiary interactions stabilizing the bent shape of the kink-turn – signature interaction (SI) and A-minor 0 are in cyan. The nucleotides are labeled according to their established annotation,^1^ which will be used throughout the study. (B) The X-ray structure of isolated Kt-7 (PDB: 4C40)^15^ with carbon-atom color-coding as in panel (A). The H-bonds are indicated by dashed lines. The base-phosphate type 4 (4BPh),^25^ sugar–phosphate, and sugar–base interactions are in yellow, and the AG base pair H-bonds are in black. The RNA backbone is highlighted with a black ribbon. The insets (i)–(v) show selected interactions in greater detail. (C) Comparison between the A-minor 0 and A-minor I tertiary interactions. The individual H-bonds constituting both types are shown.

The solution experimental data are unfortunately fairly limited for the kink-turn motif, with no NMR structures of Kt-7 available. An NMR solution structure of the “pre-formed” kink-turn U14 has been reported, featuring a fully formed SI and a water-mediated A-minor 0 interaction, though the latter interaction appears to be underdetermined by the primary experimental data.^22^ However, the sequence of this kink-turn is relatively far from consensus, and the structure lacks several typical kink-turn features. An NMR solution structure of an unfolded (unkinked) kink-turn U4 is also available, featuring fully formed C and NC stems but missing the SI and A-minor interactions.^23^ Note that most kink-turns do not kink in the absence of proteins or divalent ions; however, FRET experiments confirm that the Kt-7 is fully kinked in the presence of a rather small amount (∼70 mM Na^+^) of monovalent ions.^1,2,24^

In addition to the SI and A-minor interactions, there are additional auxiliary tertiary contacts and features characterizing the vast majority of kink-turn motifs that are exemplified in Kt-7, namely, the 4BPh base-phosphate interaction between G_3n_ and A_2b_,^25^ the sugar–base interaction between G_1b_ and G_2n_, and the sugar–phosphate interaction between A_L2_ and A_L3_ ^26^ (Figure 1B).^1^ For several of its backbone dihedral suites, Kt-7 also adopts noncanonical backbone conformations (Supporting Information Table S2) that are conserved for kink-turn motifs in general.^27,28^

Molecular dynamics (MD) simulations are a computational method for observing the thermal fluctuations (dynamics) of biomolecular systems with an essentially unlimited spatiotemporal resolution. The atomic movements are described using carefully calibrated empirical potentials, collectively referred to as force fields (FFs).^29^ Assuming a high-quality starting structure, the success or failure of the MD simulations to reproduce real biomolecular dynamics rests on the quality of the utilized FF. In the case of RNA, a persistent challenge lies in developing an FF capable of accurately describing both single-stranded and structured RNAs.^30^ Despite over a decade of dedicated efforts,^31–49^ this remains an open-ended question, with uncertainty as to whether such a comprehensive solution is even achievable.^50,51,74^ As occasionally noted earlier, the Kt-7 could be a useful benchmarking system for RNA FF testing.^47,52,53^ Its densely packed network of noncanonical interactions makes it highly sensitive to even relatively small FF imbalances, especially those arising from efforts to stabilize the single-stranded A-RNA conformations, which have been common target systems in recent RNA FF development attempts.^32,35,45,48^ This could make Kt-7 an important model for regression testing to detect potential FF overfitting.

In this work, we perform extensive benchmarking of a series of modern RNA FFs, namely, the pairwise additive OL3 (ff99bsc0χ_OL3_),^54–56^ DESRES,^40^ DES-Amber,^53^ ROC,^57^ PAK,^58^ BSSF1,^59^ Chen&Garcia,^60^ CHARMM36,^61^ and the polarizable FFs CHARMM_Drude_ ^62–65^ and AMOEBA.^66,67^ We have further tested two extensions of the OL3 FF abbreviated as OL3_0BPh,CP_-gHBfix21 and OL3_R2.7_, which are described in the Methods section. Our study unveils a very diverse performance with some FFs providing entirely stable trajectories and others leading to loss of the kinked shape (straightening) of the kink-turn. To some extent, all tested FFs struggled with one or more Kt-7 interactions, even though most preserved its kinked shape. Taken together, our data highlight Kt-7 as a very informative model for evaluating the balance of interactions in RNA FFs. The rich equilibrium dynamics of Kt-7 ^68,69^ means that many types of FF imbalances lead to relatively swift occurrence of structural distortions, well within the time scales easily accessible by standard MD simulations on contemporary GPU-accelerated hardware. This minimizes the need for long simulation trajectories or enhanced sampling methods when benchmarking Kt-7. In addition, the Kt-7 autonomously folds in the presence of monovalent ions^2,24^ and does not require Mg^2+^, whose computational modeling poses challenges due to the limited accuracy of current divalent ion models.^30,70^ In this study, we describe the key structural features that should be monitored during FF benchmarking on Kt-7. We further recommend that it could be consistently included in the model sets used for FF testing due to the many potential pitfalls in simulations and its distinctiveness from typical training sets, which primarily consist of tetranucleotides and tetraloops.^33,35^ Any new RNA FF claiming general applicability should ideally demonstrate performance on Kt-7 that matches or exceeds previous parametrizations to avoid imbalance or overfitting to specific structural motifs or interactions. Note that the comparison of the FFs based on static X-ray structure constitutes the major limitation of our work, which should be taken into consideration when assessing the simulation fluctuations and developments. Together with the structural complexity of the fold, this currently limits the usability of Kt-7 for the direct parametrization of FFs. However, due to its distinctive structural features, Kt-7 represents an important regression test for the assessment of newly parametrized FF versions.

## Materials and Methods

### Starting Structure Selection and RNA FFs

We used the X-ray structure of isolated Kt-7 (PDB: 4C40)^15^ as the starting structure for almost all our simulations. Several structures of isolated Kt-7 are available in the PDB database, all exhibiting high structural similarity (Supporting Information). Notably, the characteristic Kt-7 interactions (see above) are conserved across all of these structures (Supporting Information Table S1). The final choice of PDB entry 4C40 was guided by its decent resolution (2.2 Å) and no extensive crystallographic contacts were observed in the regions of interest. A single kink-turn molecule was extracted from the asymmetric unit and truncated as shown in Figure 1A. We propose that the truncation does not affect the simulation outcomes, as supported by control simulations on the full, nontruncated structure (Supporting Information). In fact, the kink-turn is a prominent RNA motif whose structure is dictated by its sequence, whereas its stability does not depend on the flanking helices.^1,2,10^

The tested RNA FFs included nonpolarizable FFs OL3,^54–56^ DESRES,^40^ DES-Amber,^53^ ROC,^57^ PAK,^58^ BSSF1,^59^ Chen&Garcia,^60^ and CHARMM36,^61^ as well as polarizable FFs CHARMM_Drude_ ^62–65^ and AMOEBA.^66,67^ We also tested two recently proposed modifications of the nonbonded terms in the standard OL3 FF, abbreviated as OL3_0BPh,CP_-gHBfix21 and OL3_R2.7_. The OL3_0BPh,CP_-gHBfix21 variant primarily incorporates the gHBfix21 potential, which was developed using a machine-learning approach trained on experimental data for RNA tetranucleotides and tetraloops.^47^ This potential fine-tunes H-bond interactions and was parametrized together with the OPC water model and the so-called Case phosphates (CP) modification, a refinement of phosphate oxygen Lennard-Jones (LJ) parameters introduced by Steinbrecher et al.^71^ The CP modification was applied for the OL3_0BPh,CP_-gHBfix21 in the present study, alongside our correction of the α, γ, δ, and ζ backbone dihedral potentials, which compensates for the altered phosphate LJ parameters.^72^ Additionally, we incorporated an independently developed nonbonded fix (NBfix) correction, NBfix_0BPh_, which adjusts the LJ potential for −H8···O5′ and −H6···O5′ atom pairs.^49^ This correction eliminates steric clashes in intranucleotide type 0 base-phosphate (0BPh) interactions. The OL3_R2.7_ variant applies a similar NBfix correction to almost all C–H···O interactions in RNA, universally reducing the H···O *R*_min_ LJ distance to 2.7 Å.^73^ This “hydrogen repulsion modification” was originally designed to mitigate C–H···O steric conflicts in RNA hairpin loops but it also introduces spurious C–H···O interactions in other regions of RNA structures.^74^ In addition to simulations starting from the structure of isolated Kt-7, we also tested the OL3 FF on the Kt-7 structure extracted from the *H.m.* large ribosomal subunit (residues 76–83 and 91–101; PDB: 1S72),^75^ which has the A-minor I starting conformation instead of the A-minor 0 (see Introduction).

### Water Models and Ion Parameters

When selecting the water model and ion parameters, we primarily followed the specific recommendations in the original literature made by the FF authors. When none were given, we made a choice reflecting the typical use patterns observed in various studies. To that end, we used OPC^76^ with OL3, OL3_0BPh,CP_-gHBfix21, and PAK, TIP4P-D^77^ with DESRES and DES-Amber, TIP3P^60^ with ROC, Chen&Garcia, and BSSF1, SPC/E^78^ with OL3, and the CHARMM-modified TIP3P with CHARMM36. We used the SWM4-NDP^79^ and AMOEBA^66,67^ water models for CHARMM_Drude_ and AMOEBA, respectively. The chosen ion parameters also differed depending on which FF and water model were utilized. For systems using the OPC, TIP3P, and SPC/E water models, we used the Joung&Cheatham parameters^80^ (TIP4PEW Joung&Cheatham ion parameters were used for OPC), except the OL3_R2.7_ FF where the Li&Merz parameters were used.^81^ In some simulations, we also included magnesium ions, described using the Li-Merz 12–6 parameters. The DESRES and DES-Amber simulations were run with the recommended Charmm22 ^82^ ion parameters, Chen&Garcia with Chen&Pappu^83^ ion parameters, and CHARMM36 with the CHARMM ion parameters.^84^ The CHARMM_Drude_ and AMOEBA default ion parameters were used. All of the RNA FF/water/ion parameter combinations utilized are listed in Table 1.

**Table 1:** List of the MD Kt-7 Simulations

force field	solvent model	ion parameters	number of simulations × length (μs)
***Free Kt-7***			
OL3	OPC	Joung&Cheatham	5 × 10
OL3	SPC/E	Joung&Cheatham	5 × 10
OL3^a^	SPC/E	Joung&Cheatham	5 × 10
OL3^b^	SPC/E	Joung&Cheatham	5 × 20
OL3^c^	SPC/E	Joung&Cheatham + Li&Merz (Mg^2+^)	5 × 10
OL3^c^ ^,^ ^d^	SPC/E	Joung&Cheatham + Li&Merz (Mg^2+^)	5 × 10
OL3^e^	SPC/E	Joung&Cheatham	5 × 10
OL3_0BPh,CP_-gHBfix21	OPC	Joung&Cheatham	5 × 10
OL3_R2.7_	OPC	Li&Merz	5 × 10
PAK	OPC	Joung&Cheatham	5 × 10
ROC	TIP3P	Joung&Cheatham	5 × 10
Chen&Garcia	TIP3P	Chen&Pappu	5 × 10
DESRES	TIP4P-D	Charmm22	5 × 10
DES-Amber	TIP4P-D	Charmm22	5 × 10
BSFF1	TIP3P	Joung&Cheatham	5 × 10
CHARMM36	TIP3P_Charmm36_	CHARMM	5 × 10
CHARMM_Drude_	SWM4-NDP	CHARMM_Drude_	5 × 2.5
AMOEBA	AMOEBA	AMOEBA	5 × 2.5
***Kt-7 complexed with L7Ae protein***			
OL3	SPC/E	Joung&Cheatham	3 × 10
AMOEBA	AMOEBA	AMOEBA	3 × 1

a A significantly increased KCl concentration of 1 M was used.

b Simulations were started from a Kt-7 structure excised from ribosome, which possesses the A-minor I interaction. They will henceforth be referred to as OL3­(SPC/E)-AMI.

c Instead of the standard ion concentration of 0.15 KCl, the simulations were performed at a salt concentration of 0.10 M NaCl and 0.020 M MgCl_2_ (see Methods and Supporting Information).

d Simulations were performed at the temperature of 100 K (Supporting Information).

e Simulations were performed using the nontruncated Kt-7 structure as a start (see Methods and Supporting Information).

### Simulation Protocol – the Nonpolarizable FFs

The Kt-7 structure was placed in a cubic box of water molecules with a minimal distance of 12 Å between the solute and the box border. KCl ions were added at random positions to neutralize the systems and obtain an excess salt concentration of 0.15 M. This concentration represents the standard settings commonly used in nucleic acid simulations,^30^ mimics the physiological *in vivo* monovalent ion conditions, and exceeds the minimal monovalent ion concentration at which Kt-7 has been experimentally shown to fold autonomously and without any magnesium ions present.^24,30^ Nevertheless, we note that these conditions differ from those used in the X-ray crystallization experiments of our chosen starting structure, which were performed in the presence of 0.10 M NaCl and 0.020 M MgCl_2_. Control simulations under the crystallization ion conditions confirmed that these differences do not influence the simulation outcomes (Supporting Information). The tLeap program of AMBER was used to generate the initial files with the exception of DES-Amber, BSSF1, and CHARMM36 FFs (see below). Excluding these FFs, the simulations were subsequently performed in AMBER20 ^85^ using the pmemd.MPI and pmemd.cuda^86^ programs for equilibration and production simulations, respectively. The MD simulations were run at a temperature of 298 K, with the hydrogen mass repartitioning scheme^87^ applied, allowing a 4 fs integration time step. Note that the X-ray structural data were collected at 100 K, and we therefore performed control simulations at 100 K as well (Supporting Information). Long-range electrostatics were treated with particle mesh Ewald (PME),^88^ and the distance cutoff for Lennard-Jones interactions was set to 10 Å. Production simulations were performed in a constant volume ensemble, with the temperature controlled by a Langevin thermostat.^89^ For more details of the minimization and equilibration protocols, see ref 69. The DES-Amber, BSSF1, and CHARMM36 simulations were performed in Gromacs2020.^90^ The simulation protocol in Gromacs2020 slightly differed from that in AMBER20 due to differences in the simulation codes. Specifically, Gromacs simulations were performed in a rhombic dodecahedral box and bonds involving hydrogens were constrained using the LINCS algorithm.^91^ The cutoff distance for the direct space summation of the electrostatic interactions was 10 Å and the simulations were performed using the stochastic velocity rescale thermostat.^92^ All production simulations with pair-additive FFs were run for 10 μs (the OL3 simulations of Kt-7 possessing an A-minor I interaction at the start were run for 20 μs) with five independent trajectories produced (Table 1).

### Simulation Protocol – the Polarizable FFs

Five Kt-7 structures were pre-equilibrated using the OL3­(OPC) FF and used as starting points for CHARMM_Drude_ ^62–65^ and AMOEBA^66,67^ simulations. For CHARMM_Drude_, the structures were transformed into the polarizable model using the CHARMM software (version 44b1).^84^ During the conversion process, Drude particles were introduced for all heavy atoms and lone pairs associated with each H-bond acceptor. The OPC water molecules were converted into the polarizable SWM4-NDP model.^79^ After initial minimization and equilibration procedures using the NAMD 2.13 package,^93,94^ five independent production simulations were performed at 298 K in OpenMM 8.0 ^95^ for 2.5 μs. The Drude Langevin integrator^96,97^ was utilized with a time step of 1 fs. The pressure was maintained at 1 bar utilizing the Monte Carlo barostat.^98^ Covalent bonds involving hydrogens were kept rigid using the SHAKE^99^ and SETTLE^100^ algorithms for solute and waters, respectively. A constraint of 0.2 Å was applied to limit the length of the Drude-nuclei bonds. Electrostatic interactions were treated using the PME method^88^ with a 12 Å cutoff for the real-space term. Nonbonded interactions were truncated at 12 Å, using a switching function from 10 to 12 Å.

For the AMOEBA FF, pre-equilibrated Kt-7 structures were transferred into *xyz* coordinates by Tinker and minimized in 10,000 steps using the steepest descent method. The systems were then heated to 298 K and equilibrated at a pressure of 1 bar. A stochastic velocity rescale thermostat^92^ and Monte Carlo barostat^98,101^ with coupling constants of 0.1 ps were used to maintain temperature and pressure, respectively. The applied real-space cutoffs for electrostatic and van der Waals interactions were 7 and 12 Å, respectively. The RESPA integrator^102^ was used with the integration step of 1 and 2 fs for equilibration and production simulations, respectively. Other control functions and parameters were set to their default values. We ran five independent simulations for 2.5 μs in the NVT ensemble using the GPU-accelerated 2023 Tinker-HP 1.3 code.^103^

### Simulation Protocol – the L7Ae Protein/Kt-7 Complex Simulations

The FFs with the best performance in simulations of the isolated Kt-7 (OL3­(SPC/E) and AMOEBA (see Results and Discussion) were further tested in the context of its complex with the L7Ae protein. The X-ray structure of this protein–RNA complex (PDB: 4BW0) was used as the starting point.^15^ The OL3 FF for RNA was paired with the ff14SB protein FF.^104^ Pre-equilibrated structures for the AMOEBA simulations were prepared by using the aforementioned OL3­(SPC/E)/ff14SB FF combination. All other parts of the simulation protocol mirrored those used for isolated Kt-7 (see above). Three trajectories were generated for each FF, with lengths of 10 μs for OL3­(SPC/E)/ff14SB and 1 μs for AMOEBA. The shorter time scale of AMOEBA simulations was due to the significantly larger computational costs associated with the use of polarizable FF for a system of this size.

### An Overview of Simulation Protocols

An overview of the simulation protocols and software packages applied to different FFs is provided in Supporting Information Table S3.

### Analyses of Structurally Essential Tertiary Contacts

All analyses were performed using the cpptraj module of Amber 20. To evaluate the stability of the kink-turn motif, we primarily monitored the H-bonds forming the tertiary contacts that stabilize its kinked shape. The individual direct H-bonds were considered present when the distance between heavy atoms was below 4.0 Å and the donor-hydrogen-acceptor angle was above 120°. The angle condition was not imposed on H-bonds involving the O2′-groups as it would have led to excessive false negatives due to their greater conformational flexibility. It is worth noting that the 4.0 Å heavy-atom distance is a lenient threshold that should largely encompass the natural thermal fluctuations of a common H-bond at 298 K. Nevertheless, due to the hard-limit nature of this cutoff, H-bonds with occupancies ∼90% in simulations can still be regarded as essentially fully stable. In other words, H-bond occupancies of 100% are not always to be expected, and failure to reach them in simulations is not indicative of a potential FF issue.

The first H-bond to be analyzed was the signature interaction (SI), G_L1_(O2′)-A_1n_(N1) (Figures 1B and 2A). In many simulations, SI tends to reversibly fluctuate between the *native* G_L1_(O2’)-A_1n_(N1) arrangement and the G_L1_(O2’)-A_1n_(N6) H-bond, which we call *non-native* SI throughout the paper (Figure 2B). The populations of both arrangements were evaluated. In the present study, we generally consider the SI as formed for both arrangements, albeit to our knowledge, the latter H-bond has never been observed in kink-turn experimental structures. We nevertheless consider its reversible formation as a minor structural perturbation, which does not indicate a major FF imbalance.

**Figure 2:**
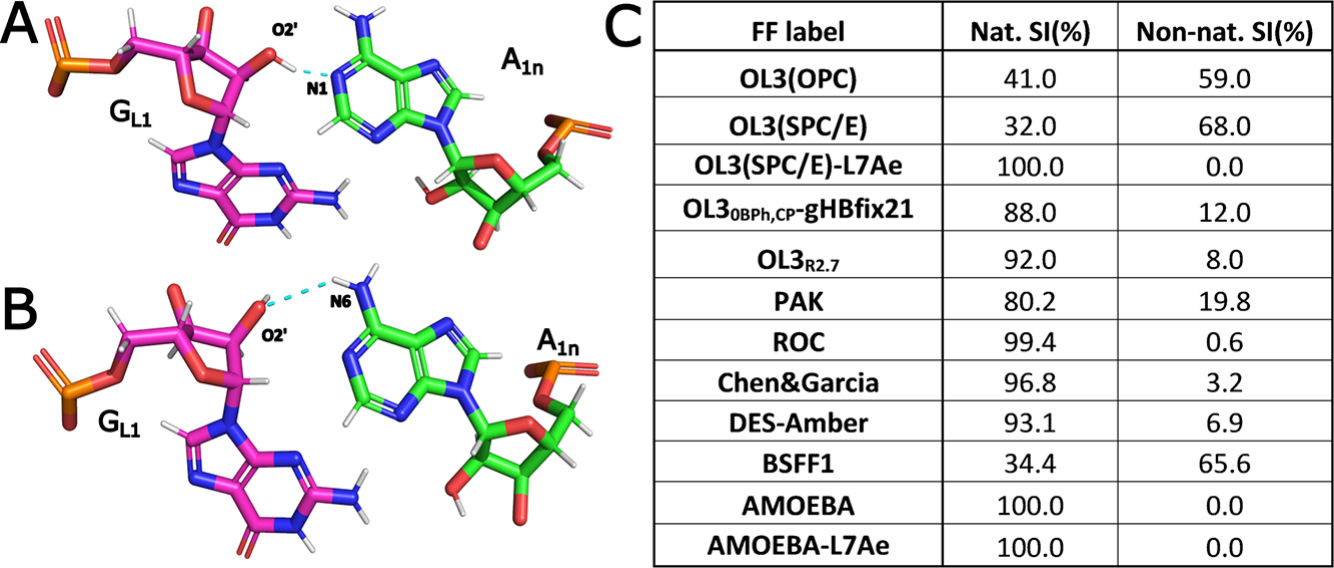
Native and non-native SI conformations observed in MD simulations of Kt-7. Snapshots from the simulations showing the (A) native and (B) non-native SI. (C) Table showing the populations of the native and non-native SI conformations in the combined simulation ensembles. In simulations where both populations existed, they regularly exchanged on the nanosecond time scale. “L7Ae” indicates simulations with the bound L7Ae protein (see further below).

The A-minor 0 interaction of the starting structure (PDB: 4C40) can sometimes transition into the A-minor I arrangement in the simulations. Therefore, we monitored the two sets of H-bonds corresponding to both A-minor types (Figure 1C)^21^: specifically, G_–1n_(O2’)-A_2b_(N3) and G_–1n_(O2’)-A_2b_(O2’) H-bonds for A-minor 0 referred to as AM0_A_ and AM0_B_, respectively, and G_–1n_(O2’)-A_2b_(N1), C_–1b_(O2’)-A_2b_(O2’), and A_2b_(N3)-G_–1n_(N2) H-bonds denominated as AMI_A_, AMI_B_ and AMI_C_ (Figure 1C), respectively, for A-minor I. When the A-minor I interaction is formed, the simulations commonly populate temporary states with a water insertion into the C_–1b_(O2’)-A_2b_(O2’) interaction. Formation of this water bridge does not constitute disruption of the structure but rather its natural dynamics; such arrangements are in fact seen also in some experimental kink-turn structures.^105,106^ Thus, our analyses considered the A-minor I formed even when this H-bond showed values above the cutoff, as long as the other two H-bonds were established.

In addition to the tertiary, SI and A-minor interactions that directly stabilize the kinked conformation of the kink-turn, the H-bonds constituting the three *t*HS AG base pairs of the NC stem, namely, the A_1n_(N6)-G_1b_(N3), G_1b_(N2)-A_1n_(N7), A_2b_(N6)-G_2n_(N3), G_2n_(N2)-A_2b_(N7), A_3b_(N6)-G_3n_(N3), and G_3n_(N2)-A_3b_(N7) (Figure 1) were also monitored. In the C-stem, we monitored H-bonds constituting the Watson–Crick base pair closest to the bulge region, namely, the G_–1n_(O6)-C_–1b_(N4), G_–1n_(N1)-C_–1b_(N3), and C_–1b_(O2)-G_–1n_(N2). This GC base pair participates in the A-minor interaction (Figure 1B).

### Additional Analyses

The following analyses were conducted exclusively on portions of the simulation ensembles where SI remained stable, as defined above. This approach was chosen to minimize noise in the data, as empirical observations showed that unstable SI essentially always implied severely disrupted kink-turn structures; the SI is the most important H-bond in the kink-turn (see the Introduction). In this fashion, we monitored the χ-glycosidic dihedral of A_L2_, with values in the range of −90° to 90° considered to be *syn* and outside of this range as *anti-*conformations. Stacking of A_L2_ with either G_L1_ or A_1n_ was evaluated by calculating the distance between geometric centers of the endocyclic N-atoms of each base. Stacking was considered present for distances below 4.0 and 4.5 Å for the A_L2_/A_1n_ and A_L2_/G_L1_ stacks, respectively. These cutoff values were chosen empirically to well reproduce the results obtained by visual analysis. The 4BPh interaction was considered present if either the A_2b_(OP2)-G_3n_(N1) or A_2b_(OP2)-G_3n_(N2) or both H-bonds were formed (see above for a general H-bond presence definition). We also evaluated the presence of the A_L3_(O2’)-A_L2_(OP1) and G_1b_(O2’)-G_2n_(N2) H-bonds, constituting the characteristic sugar–phosphate and sugar–base interactions, respectively (Figure 1B). Individual RNA acceptor atoms were considered to be binding a K^+^ or Na^+^ if the ion was located within 3.5 Å. The binding sites of the Mg^2+^ ions for the inner and outer shell binding were identified based on a cutoff of 3.5 and 4.8 Å, respectively. The density grid showing the strongest ion binding sites was visualized with VMD. Backbone dihedrals and sugar puckers^107^ were calculated with cpptraj and visualized using Python’s matplotlib module. We also monitored the correlated time development and distribution of the backbone dihedral suites^28^ exhibiting noncanonical α/γ values in the experimental structure of Kt-7, i.e., the A_1n_/G_–1n_, G_L1_/A_L2_, and A_L2_/A_L3_ suites (Supporting Information Table S2).

## Results and Discussion

### General Comments and the Global Stability of Kt-7

In the following, we describe performance of the individual FFs both in a global sense (i.e., whether the kinked shape of Kt-7 is maintained) and in the context of the individual characteristic interactions, going from the most essential to less important interactions. Out of all the structural features described below, we suggest that the ability to reproduce the SI and A-minor interactions should serve as the primary criterion for evaluating FF performance on Kt-7. Although other structural details may vary among different kink-turns, these two interactions are in some form always present.^3^ Note that there is only limited solution experimental information on kink-turns and that X-ray structures definitely have their own inherent limitations, including differences in temperature, crystal packing effects, and the presence of nonphysiological chemical agents or ions used during crystallization.^108^ The crystal environment is structurally restrictive for the RNA, and it could potentially mask minor conformational states accessible in solution. Nevertheless, we argue that an FF failing to reproduce the SI or A-minor interactions to a substantial degree, while also sometimes leading to irreversible structural degradation on the microsecond time scale of standard MD simulations, reflects deficiencies in parametrization rather than genuine dynamics of the motif in solution.

In total, we have performed 96 standard MD simulations with cumulative length of 908 μs. Multiple independent trajectories were generated using each FF (Table 1), revealing a consistent performance but relatively large variability in the time scales of specific events. In other words, while characteristic transitions or instabilities consistently occurred for a given FF, the timing of their initial occurrence varied significantly. Despite this variability, we propose that the simulations were sufficiently long to evaluate the performance of each FF. The qualitative agreement between independent trajectories suggests that the sampling, while clearly not ergodic, was sufficient to capture the dominant dynamical features relevant for FF assessment. See also the Supporting Information for block-size-averaging convergence analyses of the individual simulation ensembles. Unless otherwise specified, the observations described below were identified in all or most independent trajectories (replicates) obtained for a given FF.

Note that we observed irreversible unkinking of Kt-7 and/or serious H-bond disruptions of the noncanonical stem in at least one trajectory using the BSSF1, DES-Amber, ROC, OL3_0BPh,CP_-gHBfix21, and OL3_R2.7_ FFs (see the Supporting Information for the description of the unkinking, Tables S4 and S5). These events were rare and occurred inconsistently across trajectories, and we therefore do not interpret them as indicative of general FF failure. However, in each instance, the respective FFs also showed systematic under-representation of key stabilizing interactions (typically the A-minor interaction), which seemed to have directly triggered or accelerated the unkinking. For three FFs (CHARMM36, CHARMM_Drude_, and DESRES), the simulations did not reveal a sufficiently accurate description of the kink-turn. To streamline the presentation, these three FFs are not included in the following paragraphs analyzing the individual interactions, as we were not able to obtain reliable statistics. However, their overall performance is discussed at the end. Lastly, simulations of Kt-7 under crystallographic salt conditions or temperatures, as well as simulations of the nontruncated Kt-7 structure, are detailed in the Supporting Information.

### Kt-7 Signature Interaction (SI) Populates Two Conformations in MD Simulations

The SI is the most important and defining interaction of the kink-turn motif (Figures 1B and 2).^3^ Whenever a global loss of the kink-turn’s kinked shape (see above) occurred, the SI was typically the last tertiary interaction to disappear. Thus, the presence of the SI serves as a reliable and straightforward indicator of the overall structural integrity of the kink-turn. Although instances of temporary localized disruptions of the SI without complete unfolding of the kink-turn were observed, such events were relatively rare. As observed earlier,^68,69,109^ many of our simulations revealed the SI sampling two distinct conformations that interchanged rapidly on the nanosecond time scale. Specifically, the native A_1n_(N1)-G_L1_(O2’) H-bond has been regularly replaced with the G_L1_(O2’)-A_1n_(N6) H-bond, henceforth referred to as the native and non-native SI, respectively (Figure 2). These transitions were always reversible on the simulation time scale with all FFs that displayed them, and they never propagated to larger perturbations of the kink-turn. The non-native SI could conceivably be accessible as a minor population state in solution at room temperature, even though it is not observed in any of the available crystal structures. Therefore, in this paper, we consider both conformations to correspond to a stable SI. Still, an ideal FF should probably dominantly sample the native SI arrangement.

The individual RNA FFs exhibited strikingly different propensities to sample the two SI conformations. Specifically, the OL3 FF variants (OL3­(OPC) and OL3­(SPC/E)), as well as BSSF1, favored the non-native SI. The population of native SI is increased for the OL3_0BPh,CP_-gHBfix21 and OL3_R2.7_ modifications. The PAK, ROC, DES-Amber, and Chen&Garcia FFs also favored the native SI. Performance of the FFs favoring the native SI can at first glance look better; however, we stress that some of these FFs had significant troubles with the description of the A-minor interaction, the second major interaction stabilizing the kink-turn motif. We emphasize the nearly 100% population of the native (SI observed with both the nonpolarizable ROC and the polarizable AMOEBA FF. Notably, in the case of AMOEBA, the non-native SI was not observed at all (Figure 2C). This suggests the occurrence of the non-native SI in simulations with nonpolarizable FFs might be related to the lack of polarizability. In the case of ROC, this limitation appears to be effectively compensated for through adjustments to the backbone dihedral angles, resulting in an excellent representation of native SI by the ROC FF.

### A-Minor 0 Interaction is Problematic for Some of the Recent FFs

Essentially all tested FFs struggled to fully maintain the A-minor 0 interaction (Figures 1C and 3 and Supporting Information Table S6). To compare the performance of the FFs, we mainly considered the G_–1n_(O2’)-A_2b_(N3) H-bond interaction of the A-minor 0, which was the more stable one with all FFs and is referred to as AM0_A_. Note that the AM0_A_ is also indicated as more stable, with a significantly shorter interatomic distance, in all available experimental structures of isolated Kt-7 (Supporting Information Table S1). Thus, we suggest that the FFs should optimally sample the AM0_A_ to a high degree. We used the relative stability of the second interaction defining the A-minor 0 motif,^21^ namely, the G_–1n_(O2’)/A_2b_(O2’) (AM0_B_), as an auxiliary measure. In most X-ray structures of Kt-7, the AM0_B_ interaction exhibits an unusually long interatomic distance (Supporting Information Table S1), falling outside the typical range characteristic of direct H-bonds yet being too short to accommodate water mediation. We tentatively attribute this to structural averaging between two conformational states present in the crystal, a common limitation inherent to static X-ray structures. Indeed, in our simulations, we observed either a direct AM0_B_ interaction or a water-mediated one but never the crystallographic geometry. Due to this experimental uncertainty, we base our FF ranking on the AM0_A_ interaction, for which unequivocal experimental evidence exists in all available X-ray structures of Kt-7. Based on this evaluation, the Kt-7 A-minor 0 interaction was best reproduced by the standard OL3 variants (OL3­(SPC/E) and OL3­(OPC)), which nearly always maintained the AM0_A_ while at the same time also populating the auxiliary AM0_B_ to a high degree (Figure 3B). The PAK, AMOEBA, and ROC FFs also showed excellent results. Meanwhile, BSFF1, DES-Amber, and Chen&Garcia FFs showed relatively low populations for both AM0_A_ and AM0_B_. Interestingly, the OL3 variants that included the recently proposed NBfix_0BPh_,_CP_-gHBfix21 or R_2.7_ FF modifications performed worse compared with the unmodified OL3 variants. We note that a fully water-mediated variant of the A-minor 0 interaction has been suggested by NMR in solution for a pre-formed structure of the kink-turn U14.^22^ No such experimental evidence exists for Kt-7, which in addition has a very different sequence. The disruptions of AM0_A_ observed in our simulations are also distinct from the water-mediated state reported for U14. Nevertheless, we cannot categorically rule out that some of the A-minor 0 disruptions seen in our simulations may correspond to a so far experimentally uncharacterized “pre-formed” conformational state of Kt-7, particularly those occurring with the standard OL3 variants, as well as the PAK, AMOEBA, and ROC FFs. In contrast, the A-minor disruptions observed with the other force fields (BSFF1, DES-Amber, and Chen&Garcia) are more severe, occasionally leading to irreversible unkinking or even stem disruptions (see Supporting Information Tables S4 and S5).

**Figure 3:**
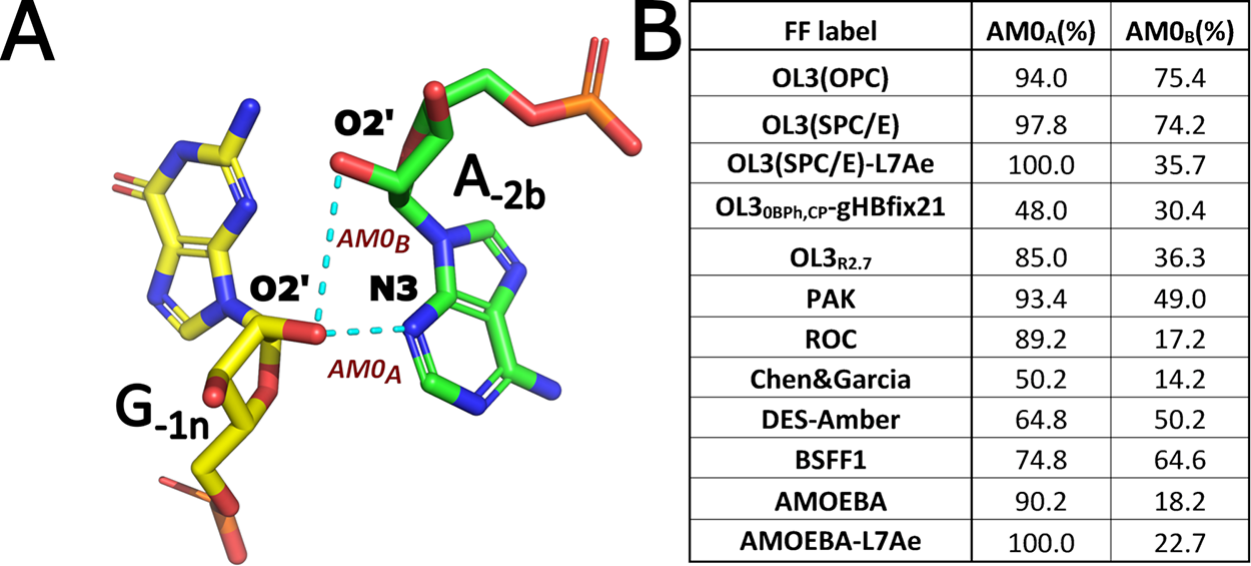
A-minor 0 interaction as observed in the MD simulations of Kt-7. (A) Structural overview of the interaction, with the H-bonds characterizing the A-minor 0 motif^21^ indicated as AM0_A_ and AM0_B_. (B) Table showing the populations of these two H-bonds in the combined simulation ensembles.

Finally, both reversible and irreversible transitions from the A-minor 0 to the A-minor I interaction could sometimes be observed with specific FFs (OL3_0BPh,CP_-gHBfix21, Chen&Garcia, ROC, and AMOEBA). This was highly correlated with stability changes of some characteristic interactions and sugar pucker populations. As the A-minor transitions occurred inconsistently across the simulation replicates, we have decided not to interpret them in terms of FF performance. However, the additional complexity these transitions pose needs to be taken into account when using Kt-7 as a model to evaluate FFs (see Supporting Information for further details).

### The Base Pairs of the NC- and C-Stems

The GC base pairs of the C-stem and the AG base pairs in the NC stem, both of which are characteristic features of the kink-turn motif, were generally stable in the simulations. In the case of the AG base pairs, there were some reversible disruptions observed in response to fluctuations of the SI interaction. This was especially the case with the AG base pair closest to the bulge (which also acts as the acceptor for the SI) where mainly the A_1n_(N6)-G_1b_(N3) H-bond could fluctuate. Fluctuations of the N6–N3 H-bonds were observed also for the other two AG base pairs but with much lower frequency and range. The N2–N7 H-bonds were in general slightly more populated than the N6–N3 H-bonds and minor differences among the FFs were observed (Supporting Information Table S7). In agreement with experiments and previous MD simulations,^15,110^ we have observed that the presence of the A-minor I interaction (see above and Supporting Information) is straining the geometry of the first AG base pair, making the N6–N3 H-bonds longer or even disrupted.

### The A_L2_ Transitions to Non-native *anti-*Conformation and Adopts an Alternative Stacking Arrangement

The second base of the bulge (A_L2_) possesses a *syn-*conformation of its N-glycosidic dihedral angle in the starting structure. This is the case for all Kt-7 structures available in the PDB (Supporting Information Table S1), as well as most other kink-turns when the second bulge nucleotide is a purine, and it can be considered a characteristic feature of the kink-turn motif.^111^ When bound to the L7Ae protein, *syn*-specific protein-RNA interactions involving A_L2_ are formed. Surprisingly, in virtually all simulations using the nonpolarizable FFs, the A_L2_ base consistently transitioned into the *anti-*conformation (Figure 4 and Supporting Information Table S8). We initially suspected an influence of crystal packing, as there are some contacts between the A_L2_ base and a neighboring molecule in the 4C40 X-ray structure. However, structural analysis of multiple isolated Kt-7 structures where the A_L2_ base is also in *syn* with no such crystal packing contacts seems to disprove this hypothesis (see Supporting Information). Only a few very short-lived returns to the *syn* state were observed in simulations, and the transition can be considered essentially irreversible. The only exceptions were the PAK and AMOEBA FFs, which allowed some reversible *syn/anti* dynamics of A_L2_. However, the *anti-*conformation was still dominantly sampled.

**Figure 4:**
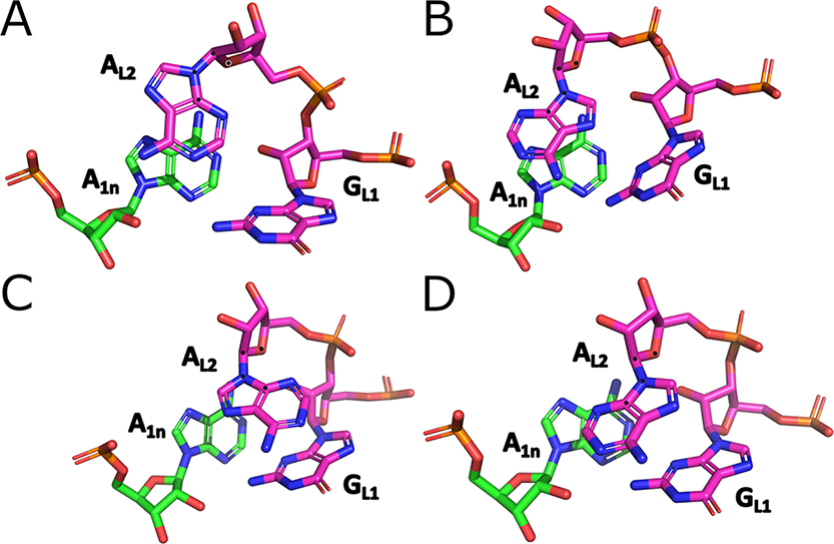
Stacking patterns and conformational states observed for the A_L2_ base. (A) A_L2_ in *syn*, stacking with A_1n_ (the native, i.e., crystal, arrangement); (B) A_L2_ in *anti*, stacking with A_1n_; (C) A_L2_ in *syn*, stacking with G_L1_; (D) A_L2_ in *anti*, stacking with G_L1_. The atoms defining the N-glycosidic dihedral angle of A_L2_ are marked with black dots.

In addition to the *syn*/*anti* transitions, A_L2_ also showed alternative stacking patterns. Specifically, A_L2_ stacked with the A_1n_ in the starting structure while all FFs except AMOEBA preferred an alternative stacking arrangement of A_L2_ with G_L1_ (Figure 4 and Supporting Information Table S8). The formation of the alternative stacking pattern appears to be independent of the *syn–anti* transitions. However, we noticed that the stacking pattern influences the sugar pucker conformation of A_L2_. Namely, the native C2’-endo sugar pucker shifts to the O4’-endo when A_L2_ begins stacking with G_L1_ while *syn* is still present. Later, as the *syn*-conformation transitioned to *anti*, the pucker changed to C3′-endo (Supporting Information Figure S1).

### The 4BPh and Sugar–Phosphate Interactions Disappear with Most FFs

The A_2b_(OP2)-G_3n_(N1/N2) 4BPh interaction (Figure 1B), another characteristic feature of the kink-turn motif, disappeared shortly after the start of simulations with all nonpolarizable FFs (Supporting Information Table S8). Once lost, this interaction was rarely restored for more than a few nanoseconds. It was, however, often reestablished when the A-minor I state was present (see the Supporting Information). The exception to the generally poor reproduction of the 4BPh interaction was the AMOEBA FF, which maintained the interaction in over 40% of the whole simulation ensemble. In the X-ray experimental structures, the 4BPh interaction is universally present (Supporting Information Table S1).^15^ Note that in solution, minor populations lacking the 4BPh interaction could realistically be expected; however, this interaction is essentially inaccessible in most simulations once it is lost. As such, we consider its swift and permanent loss as a potential FF-related problem, essentially for all FFs except for AMOEBA. We hypothesized that the stability of the 4BPh interaction might be influenced by the salt conditions. However, neither increasing the monovalent ion concentration nor including magnesium ions stabilized the 4BPh interaction, nor did these conditions produce any other detectable effects on the monitored interactions, at least on the time scale of our simulations (Supporting Information Tables S6–S9).

We also monitored the sugar–phosphate and sugar–base interactions native to Kt-7, the A_L3_(O2’)-A_L2_(OP1) and G_1b_(O2’)-G_2n_(N2) H-bonds, respectively. The sugar–phosphate interaction was typically lost shortly after the simulation start for all FFs, with the notable exception of the polarizable AMOEBA FF. This loss appeared to be coupled with the A_L2_ base transitioning from the *syn*- to *anti-* conformation, a feature common to all tested FFs (see above). The second interaction was stable across all simulations except for the DES-Amber and OL3_0BPh,CP_-gHBfix21 FFs. Temporary disruptions of this interaction could be observed in response to changes elsewhere within the kink-turn, such as A-minor 0 to I transitions, shifts in the stacking pattern of A_L2_, or changes in the sugar pucker of G_1b_ (Supporting Information Figure S2).

### Binding of the L7Ae Stabilizes the Kink-Turn’s Signature Interaction

To further explore the structural dynamics of Kt-7, we took two tested FFs (OL3­(SPC/E) and AMOEBA) and carried out simulations with the bound L7Ae protein (see Methods). In the case of OL3­(SPC/E), the binding of the protein fully stabilized the native arrangement of the SI, contrasting the simulations of the isolated Kt-7 (Figure 2C). The A-minor 0 was also fully stable with both FFs (Figure 3B, Supporting Information Table S10). The A_L2_ base remained in the *syn*-conformation in all simulations with the bound protein as it was engaged in hydrophobic contacts with the protein, blocking any potential transitions to the *anti-*conformation. Surprisingly, with OL3, we observed the 4BPh interaction being more populated in simulations of the protein–RNA complex than in the isolated Kt-7, despite this interaction being present and absent in the starting structures of the isolated and protein-bound kink-turns, respectively. With AMOEBA, we observed the opposite trend (Supporting Information Table S10). Lastly, the protein–RNA interface interactions generally fluctuated more with the AMOEBA FF, and we observed increased dynamics of the entire protein–RNA interface. However, these fluctuations were entirely reversible, and the complex interface can thus be considered as fully stable for both tested FFs.

### The FFs Somewhat Struggle to Reproduce the Noncanonical α/γ Backbone Dihedral Suites of Kt-7

We have focused our analysis of the RNA backbone dihedrals on three sugar–phosphate backbone segments (so-called suites)^28^ exhibiting characteristic noncanonical α/γ states. These include gauche+/trans (g+/t) state for suite A_1n_/G_–1n_, g+/g- for G_L1_/A_L2_, and g+/g+ for A_L2_/A_L3_ (Figure 5, Supporting Information Table S2). These backbone states are highly conserved for the kink-turn motif in general^28^ and their reproducibility by the FFs is another benchmarking opportunity, as the dihedral potentials are a common target in FF reparametrization efforts. For instance, the g+/t combination for α/γ has been explicitly penalized by most AMBER-derived FFs since the time of the parmbsc0 modification.^55^ Indeed, even though the native g+/t state of suite A_1n_/G_–1n_ was not completely eliminated, a significant population of the non-native canonical A-RNA values was present with half of tested FFs. The best performance was seen with the OL3­(SPC/E), Chen&Garcia, PAK, DES-Amber, ROC, and AMOEBA FFs where only minor populations of the non-native canonical A-RNA values were observed. There is a surprising difference between the OL3­(SPC/E) and OL3­(OPC) simulations, hinting at a potential importance of the water model choice (Supporting Information Figures S3 and S4). For the G_L1_/A_L2_ suite, the native g+/g- combination immediately and permanently transitioned into canonical A-RNA values (g-/g+) for all tested FFs except for the AMOEBA FF and the protein–RNA complex simulations. Lastly, for the A_L2_/A_L3_ suite, the native g+/g+ combination was correctly reproduced by all tested FFs with only minor canonical A-RNA populations observed (Supporting Information Figures S3 and S4). The present results confirm earlier observations that it is challenging to describe the noncanonical α/γ states in RNA by MD.^112^

**Figure 5:**
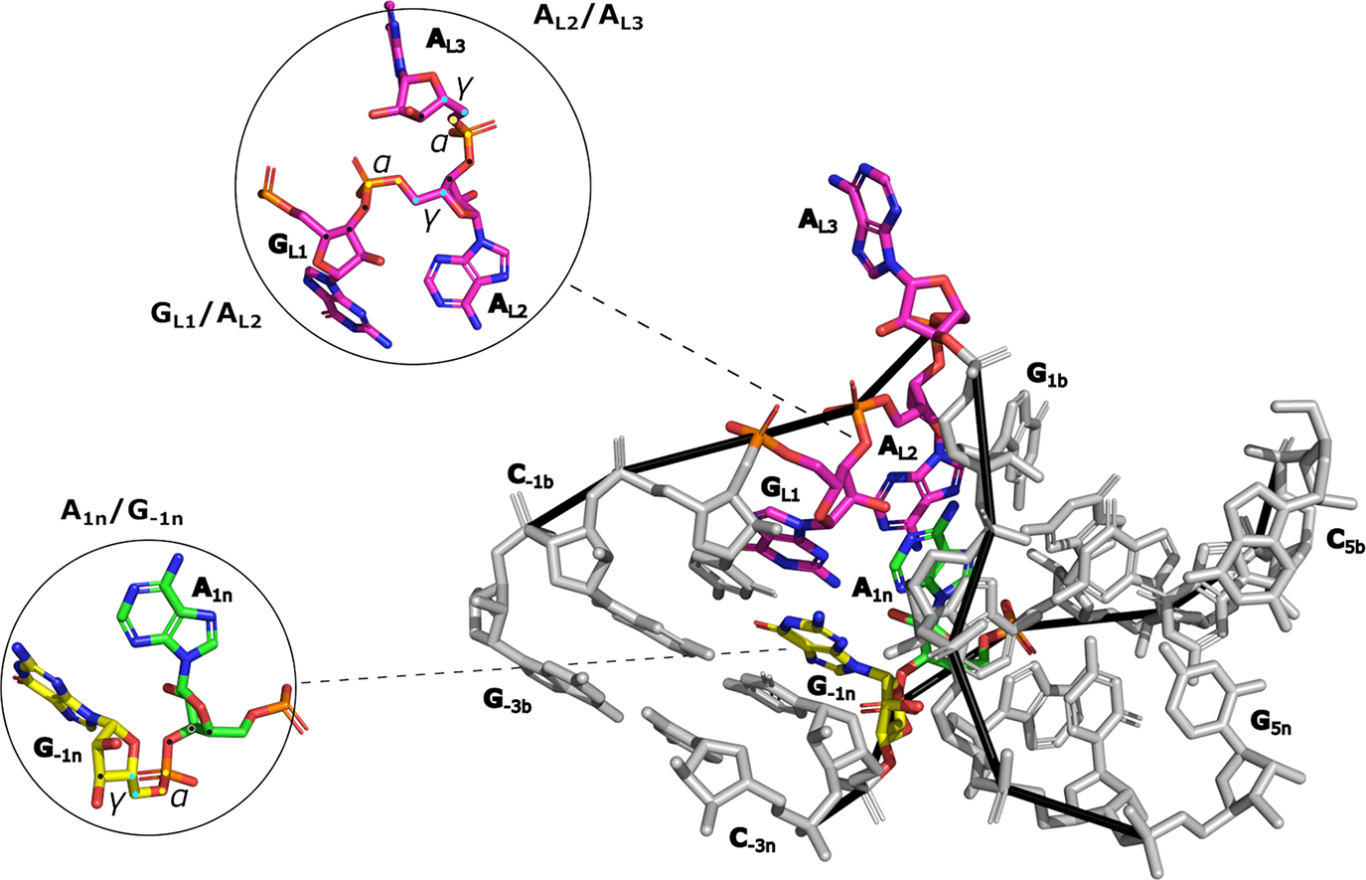
Noncanonical α/γ backbone dihedral suites of Kt-7. Kink-turn residues involved in the suites are colored as in Figure 1 while the remaining residues are gray. The insets show each suite in detail. The central atoms defining the α/γ dihedrals are colored yellow and cyan, respectively. The other atoms of the suites are indicated with a black dot.

### Additional Comments on the FF Performance on Kt-7

As noted at the beginning, we were unable to obtain a single Kt-7 trajectory using DESRES, CHARMM36, and CHARMM_DRUDE_ FFs in which the major characteristic interactions (SI and A-minor) would be consistently present for sufficiently long intervals. As a result, these trajectories could not provide sufficient statistics for the detailed assessment of the individual kink-turn features (see above). Nevertheless, even these trajectories were carefully monitored and analyzed. In the case of DESRES, we observed gradual loss of the kink-turn’s characteristic interactions and often complete loss of the kinked shape (Supporting Information Tables S4 and S5 and Figure S5). It is in agreement with previously conducted simulations of the Kt-7 starting from the A-minor I conformation.^69^ We emphasize that the simulation performance for Kt-7 was significantly improved with the DES-Amber FF (a reparameterization of the original DESRES FF).^53^ However, as we have shown above, DES-Amber still struggled with describing the A-minor interaction, populating it to a smaller degree than OL3 and most other FFs (see Figure 3B). This observation is consistent with another recent study where Kt-7 possessing an A-minor I interaction was used as starting structure.^74^ In the present work, we even observed irreversible loss of the kinked shape in one of the DES-Amber simulations (Supporting Information Table S4). It should be noted, however, that single unkinking or stem disruption events occurred also with some other FFs (Supporting Information Tables S4 and S5). Thus, such isolated incidents should not be used as indication of poor FF performance, especially since the correct balance between the kinked and unkinked populations, and kinetics of their transitions are not known.^113^ Likewise, some transient disruptions of the A-minor interaction could reflect a so far experimentally unobserved solution behavior of Kt-7, as seen for another unrelated kink-turn using NMR.^22^ Disruption of either stem, however, can be considered an FF failure, as NMR observations (for another kink-turn) point to unkinking but not to rearrangements of base pairing or stem disruptions.^23^

Large structural changes occurred with the CHARMM36 FF, where we observed swift loss of the interactions characterizing the kink-turn motif after the simulation start, shortly followed by partial or even complete disruption of both stems (Supporting Information Tables S4, S5, and S9 and Figure S7). This corresponded to a loss of the entire structure, preventing us from deriving any useful information from the CHARMM36 simulations. The problems of the CHARMM36 FF to describe the stems are consistent with our recent study on the UUCG tetraloop.^74^ In contrast, the polarizable CHARMM_DRUDE_ FF showed markedly better performance compared to the nonpolarizable CHARMM36, with some interactions of the Kt-7 being excellently reproduced (e.g., the 4BPh and sugar–phosphate interaction). The reason why we ultimately excluded the CHARMM_DRUDE_ simulations from our main analyses was its inability to maintain the A-minor interaction, which was universally lost soon after the trajectory start and never reformed in any trajectories except for a few short fluctuations (Supporting Information Table S6). Subsequently, the CHARMM_DRUDE_ FF struggled with reproducing the SI interaction as well (Supporting Information Table S9). Nonetheless, the benefit of explicit polarization was quite apparent as CHARMM_DRUDE_ successfully reproduced interactions and conformations where the AMOEBA FF as well showed improvements over the nonpolarizable FFs (e.g., A_L2_ in *syn*-conformation, the 4BPh interaction and the sugar–phosphate interaction, Supporting Information Table S8). The markedly different performance of the two polarizable force fields for the A-minor and SI interactions may stem from their distinct design philosophies. Specifically, AMOEBA is based on induced dipole moments, whereas CHARMM_DRUDE_ employs a classical Drude oscillator model.^62,66^ We also note that the AMOEBA FF includes atomic dipoles and quadrupoles, which might improve the description of H-bonds. Nevertheless, it is clear that polarizability could be important for the description of systems such as Kt-7, making it a useful benchmark system for refining polarizable FFs as well. Unfortunately, without adequately reproducing the critically important A-minor interaction and SI, CHARMM_DRUDE_’s performance for Kt-7 cannot be deemed satisfactory at the moment. For some other recent benchmarks of the RNA CHARMM_DRUDE_ FF, see refs 74,94,114,115 .

In conclusion, among the tested FFs, OL3­(SPC/E), OL3­(OPC), and AMOEBA showed the best overall performance for Kt-7. The polarizable AMOEBA was especially notable in maintaining some of the native interactions, which were lost with all the other FFs. We, however, note that simulations with the AMOEBA and CHARMM_DRUDE_ FFs were four times shorter compared to simulations using the nonpolarizable FFs. Nevertheless, all of the FF problems described above tended to occur relatively early in simulations. Therefore, we suggest the good performance of AMOEBA FF to be conclusive. From the nonpolarizable FFs, we wish to emphasize the good performance of the OL3­(SPC/E) and OL3­(OPC) variants, i.e., the standard OL3 RNA FF (see Methods). In particular, the OL3 FF described the A-minor interaction, which is a frequent point of failure, best among the tested FFs. ROC and PAK were the other nonpolarizable force fields that showed overall good performance, maintaining both the native SI and the A-minor interactions to a reasonable degree, as well as preserving the noncanonical backbone dihedrals satisfactorily. Lastly, see the Supporting Information for a summary of the FF issues observed in simulations of Kt-7 and suggestions for their potential resolution during the parametrization process.

## Conclusions

We present a large-scale benchmark MD simulation study of kink-turn 7 (Kt-7), a so-called consensual kink-turn and an excellent representative example of this recurrent RNA motif, using a broad range of force fields (FFs). None of the tested RNA FFs provide a perfect description of Kt-7, highlighting the challenge of accurately balancing its diverse and interdependent structural features in contemporary MD simulations. Particularly concerning are the difficulties seen with the recently developed FFs such as DES-Amber and OL3_0BPh,CP_-gHBfix21, which otherwise improve simulations of simpler motifs like tetranucleotides and tetraloops. This underscores the risks of overfitting FF parameters to homogeneous training sets. Several more ″general″ FFs, such as basic OL3, ROC, PAK, and AMOEBA, performed better for Kt-7. Among the tested FFs, the polarizable AMOEBA model frequently emerged as a positive outlier for several structural features of Kt-7, suggesting that explicit treatment of polarizability may benefit systems like Kt-7. However, to further illustrate the complexity of RNA FF development and testing, we note that both ROC and AMOEBA FFs were shown to irreversibly disrupt the native fold of the UUCG RNA tetraloop, which is a notoriously challenging system also for the OL3 FF.^74^ In other words, the ranking of FFs based on simulations of the UUCG tetraloop and Kt-7 differ significantly. This variation in benchmark results emphasizes the necessity of testing FFs across a diverse set of RNA motifs to substantiate claims of general improvements.

The quality of the reference structures used for testing is also an important factor. For X-ray structures, common limitations include differences in temperature, crystal packing forces, and the presence of nonphysiological chemical agents or ions used during crystallization.^108^ Such factors can stabilize conformations that deviate from those found in solution or suppress minor populations of these conformations. There is unfortunately no solution structure of Kt-7 available at the moment; however, comparison across different X-ray experimental structures of Kt-7 revealed a very consistent picture of molecular interactions. Still, the use of a static X-ray structure as the benchmark is obviously the main limitation of this study.

We propose that Kt-7 should serve as an important part of the set of benchmarking structures in future RNA FF parametrization efforts, as it meets several desirable criteria: (1) Computational feasibility – its relatively small size keeps computational costs manageable, even for polarizable FFs. (2) Structural complexity – its rich network of noncanonical interactions rigorously tests FF accuracy, particularly the SI, A-minor and other tertiary H-bonding. (3) Well-defined stability metrics – its loss of the stabilizing tertiary interactions tends to progress to loss of the folded (kinked) shape, even in relatively short standard simulations. For completeness, we note that an additional test for FFs could involve modeling the free-energy landscape of Kt-7’s opening-closing dynamics or the A-minor transitions. However, this would require sophisticated enhanced sampling simulations. Our preliminary but extensive attempts at such free-energy simulations revealed severe challenges in defining a suitable simulation protocol, and the results will be published separately.

In summary, the folded structure of Kt-7 represents a useful test system. With moderate computational effort, it enables a comprehensive assessment of many aspects of RNA FFs, making it an essential benchmark for future developments in the field and a valuable complement to existing model systems. Its complexity makes it a vital regression test for avoiding overfitting and ensuring that future parameter sets remain broadly transferable across RNA architectures. However, we reiterate that Kt-7 should only be part of a broader set of training and benchmarking structures. An FF modification based primarily on the kink-turn motif would likely have a limited general applicability as well.

## Supplementary Material



## Data Availability

All raw MD data are available on the Lexis platform (https://lexis.portal.tech) within the EXA4MIND Research Project directory ‘exa4mind_wp4’ and were also uploaded to Zenodo (10.5281/zenodo.15357199).

## Notes

Comments on other Kt-7 structures in the PDB database, description of unkinking and A-minor transitions, ion binding analysis, sugar pucker analysis, description of simulations performed under experimental solvent conditions and on the nontruncated Kt-7 structure, block averaging analysis, additional comments on the FF performance and suggestions for refinement, Supporting Information figures and tables (PDF)
