# Supplementary material for: The Kink-Turn 7 Motif: An Additional Test for RNA Force Field Performance
Source: J Chem Theory Comput. 2025 Dec 8;21(24):12796–809. doi: 10.1021/acs.jctc.5c00776 (PMC12746442; doi:10.1021/acs.jctc.5c00776)
Supplement: Supplementary file 1 [file ct5c00776_si_001.pdf]

The Kink-Turn 7 Motif: An Additional test for RNA Force Field Performance

Toon Lemmens[1,2], Vojtěch Mlýnský[1], Jiří Šponer[1,3], Martin Pykal[3], Pavel Banáš[3], Michal Otyepka[3,4], and Miroslav Krepl[1,3]*

[1]Institute of Biophysics of the Czech Academy of Sciences, Královopolská 135, 612 00 Brno, Czech Republic

[2]National Centre for Biomolecular Research, Faculty of Science, Masaryk University, Kamenice 5, 625 00 Brno, Czech Republic

[3]Czech Advanced Technology and Research Institute, CATRIN, Palacký University, Křížkovského 511/8, Olomouc 779 00, Czech Republic

[4]IT4Innovations, VSB-Technical University of Ostrava, 17. listopadu 2172/15, 708 00 Ostrava-Poruba, Czech Republic

*Corresponding author: Miroslav Krepl; E-mail: krepl@ibp.cz

Supporting Information

Comparison of the 4C40 structure with other Kt-7 structures in the database. Structural analysis of the isolated Kt-7 structures available in the database (PDB entries 4CS1, 5FJ0, 5FJ1, 7EFG, and 7EFH) and their comparison with 4C40 (our starting structure in MD simulations; see the main text) revealed virtually identical structures in terms of the RMSD, the presence of the characteristic interactions (SI, $AM0_A$, $AM0_B$, 4BPh, sugar-phosphate, and sugar-base interactions), the N-glycosidic dihedral angle of A_{L2}, the backbone suites, and the sugar puckers (Tables S1 and S2). A notable feature is also the long $AM0_B$ distance in five structures (Table S1), which is in more detailed in the main text.

Unkinking of the kink-turn and the additional disruptions which may indicate FF problems. In the main text, the term *unkinking* refers to the loss of the SI and A-minor interactions, followed by straightening of the Kt-7 structure, as shown in Figure S5. All the unkinking events observed in our simulations are listed in Table S4. Although unkinking is irreversible in our simulations, it does not necessarily imply a FF problem if it occurs in just one replicate for a given FF. The kink-turn obviously must exist in some equilibrium with its unkinked conformation and the timescale of our simulations might simply not be sufficient to capture the reverse process. However, in the vast majority of cases, we observed that the unkinking leads to additional structural changes, some of them rather severe. These are likely already reflecting FF problems. The unkinked structure of the spliceosomal U4 RNA kink-turn (Kt-U4) was solved by NMR in solution.[1] In contrast to Kt-7, Kt-U4 is unkinked in presence of monovalent ions. The unkinked conformation does not show any other structural changes besides mere unkinking of the two stems. In contrast, the only two simulations of Kt-7 with unkinking where no other structural changes occurred were ROC (R1) and DESRES (R3). However, in case of the latter this was mostly due to the unkinking occurring at the very end of the simulation which left no time for other changes. Other DESRES replicates where unkinking occurred more swiftly revealed severe disruptions (Table S4 and S5). A minor additional change after unkinking occurred in the $CHARMM_{DRUDE}$ (R1), which showed the disruption of the first AG base pair (A_{1n}–G_{1b}). This base pair was previously identified as highly sensitive to fluctuations of the SI. The $CHARMM_{DRUDE}$ FF is unable to correctly describe the A-minor interaction and reproduces the SI poorly as well (see the main text), which could also be affecting the unkinking process.

More serious changes occurred in $OL3_{0BPh,CP}$-gHBfix21 (R2), DESRES (R2, R5), and DES-Amber (R4) simulations. In these simulations the A_{1n}–G_{1b} base pair was disrupted shortly after unkinking. It was then replaced by a spurious, non-native AG base pair formed between A_{1n} and G_{L1} (Figure S6). This prevented reformation of the native AG base pair and almost certainly precluded any chance for restoring the kink-turn even on a hypothetically longer timescale as the critical SI interaction could not reform under such circumstances. This simulation development is also not supported by any experimental data.

Lastly, we observed extensive degradation of the kink-turn structure in the $OL3_{R2.7}$ (R4) and DESRES (R1) simulations, where we evidenced disruption of both the Hoogsteen and Watson–Crick H-bonds of the non-canonical stem. Such large-scale loss of the structure on relatively short timescales should most likely be considered FF issues. For the sake of completeness, we also note that with the CHARMM36 FF, both stems began losing cohesion swiftly after unkinking (Figure S7). Finally, in BSSF1 (R1) and DESRES (R4), disruption of the stems occurred even without any unkinking. All the stem disruption events are listed in Table S5.

A-minor 0 ⇆ A-minor I transitions add complexity to the simulations and their analyses. The Kt-7 structure can adopt both A-minor interaction types 0 and I (the main text Figure 1C; see also the main text Introduction). While the type 0 interaction is favored in isolation and/or when the Kt-7 is bound to the L7Ae protein, the type I is observed when the Kt-7 is embedded in the ribosomal context (see also the main text).[2] Consequently, if a simulation starts with Kt-7 excised from the ribosome, an eventual transition from A-minor type I to type 0 is to be expected. Indeed, previous simulation studies utilizing such starting structure universally observed A-minor transitions from type I to 0.[3, 4] We propose that A-minor transitions could potentially be observed also when starting simulations from the structure of isolated Kt-7 with the A-minor 0 interaction formed (this study). There is currently no experimental information regarding the population balance or the expected timescale of the interconversion dynamics, and we therefore do not use these processes to evaluate FF performance. In other words, the presence or absence of such interconversion for a given FF should not be interpreted as an indicator of its quality as the transitions lack sufficient statistical weight. In addition, the interconversion dynamics is slow and constitutes a rare event, even in the trajectories where it is observed. Consequently, we only record whether such A-minor transitions occur for a given FF and note their impact on the surrounding interactions.

We regularly observed reversible A-minor transitions from type 0 to I with the $OL3_{0BPh,CP}$-gHBfix21 and Chen&Garcia FFs. In only one trajectory, a single irreversible transition from A-minor 0 to I was observed for both the AMOEBA and ROC FFs (Table S11). The A-minor transitions were not observed with other FFs in this study, which all kept the starting A-minor 0 conformation of the isolated Kt-7 (see Introduction and Methods). However, as already mentioned, when starting a simulation from the ribosomal Kt-7 structure, an eventual A-minor I to 0 transition is expected. It could serve as an additional test for the FF performance, i.e. to see whether and how the FF handles this transition. To demonstrate this, we conducted 20-μs-long simulations of Kt-7 excised from the ribosome using the best-performing pair-additive OL3(SPC/E) FF variant. In four simulations, the A-minor I successfully transitioned to A-minor 0 on a timescale ranging from 1.57 μs to 4.17 μs. The transitions were irreversible, except for one replicate where the A-minor I briefly returned (between 1.57 and 1.78 μs) but then switched again to A-minor 0 (Table S11). No transition was observed in the fifth replicate on the 20-μs-timescale, illustrating the spontaneous and rare nature of this process. The transition mechanism was the same in both directions, occurring on nanosecond timescale, with the adenine sliding

along the edge of the GC base pair without any other visible conformational changes in the core region of the kink-turn.

It is notable that we observed different stabilities of the other kink-turn interactions depending on the state of the A-minor interaction. For instance, for the OL3(SPC/E) FF, the native SI conformation was strongly favored in presence of A-minor I whereas the non-native SI prevalent in the ensemble in presence of A-minor 0 (Table S12 and the main text Figure 2). Furthermore, the 4BPh was often re-established upon formation of the A-minor I state which suggests a potential conflict among individual interactions within the kink-turn structure. Indeed, the presence of A-minor I highly correlated with the stability of the 4BPh interaction in OL3(SPC/E)-AMI, $OL3_{0BPh,CP}$-gHBfix21, ROC and AMOEBA simulations (Table S12). Finally, A-minor transitions influenced sugar pucker preferences of G_{2n}. The native C2'-endo conformation was favored in the presence of A-minor 0, whereas C3'-endo predominated for A-minor I interaction (Figure S8).

Ion binding sites of Kt-7. Occupancies of ion binding sites across all Kt-7 simulations are summarized in Table S13, revealing some notable differences among the tested FFs. Interestingly, OL3(SPC/E) simulations exhibit significantly more extensive ion binding than OL3(OPC) simulations. This highlights how the quantitative picture of ion binding is influenced by the balance of solute, water, and ion FF parameters. Although a more detailed analysis is beyond the scope of this study due to the lack of relevant experimental data for comparison, we have presented the most noteworthy findings.

The most prominent monovalent cation binding sites were observed near the Hoogsteen edges of residues G_{2n}, G_{3n} and G_{4n} (Table S13 and Figure S9), in agreement with the available kink-turn experimental structures which usually indicate magnesium binding in this region.[5] There were only negligible variations among the positions of the ion binding sites observed between the individual FFs. Instead, the differences were in the populations, albeit still minor. Specifically, we noted a slightly lower overall ion binding propensity with the polarizable FFs. In addition, some FFs (Chen&Garcia, DES-Amber and BSSF1) preferred a different order of the most occupied ion binding sites compared to the rest of the FFs, however, the effect appeared to be rather limited (Figure S9 and Table S13).

Adding the L7Ae protein generally lowered the K^+ binding at the G_{2n}(O6) and G_{3n}(O6) sites in case of the OL3(SPC/E) simulations. We suggest this to be the result of competition between the Lys56 side chain and the cation binding as Lys56 is involved in transient H-bond formation with G_{2n}(N7) (total population 19.7 %). Even when the H-bond was not formed, the lysine side-chain was still present nearby, influencing the electrostatic interactions in this region and likely repelling the cations (Tables S14 and S15). Interestingly, this effect was far less pronounced with the polarized AMOEBA FF where there were many instances of the lysine side chain and K^+ binding to the G_{2n}(O6) atom simultaneously. Such simultaneous binding was never observed with OL3 and likely reflected the explicit inclusion of polarization of the K^+-G_{2n}(O6) and lysine-G_{2n}(O6) interactions by the AMOEBA FF.

Finally, in simulations where we utilized the exact X-ray experimental salt concentration conditions, i.e. the excess salt concentrations of 0.10 M NaCl and 0.020 M $MgCl_2$, the ion binding site near the Hoogsteen edges of residues G_{2n} and G_{3n} were occupied with monovalent Na^+ cations, confirming the results of the primary simulations performed with the 0.15 M KCl excess-salt. The overall occupancy of Na^+ cations was lower than that of K^+ cations, presumably due to their lower number in the simulation box. We did not observe significant binding of Mg^{2+} at these ion binding sites that would compete against the sodium. Instead, they preferred interacting with the negatively charged phosphate groups, most significantly near the bulge where the backbone is tightly kinked, which positions multiple phosphate groups in close proximity (Table S16).

The non-canonical C2′-endo sugar puckers. In the main text, we discussed several backbone suites of Kt-7 possessing non-canonical α/γ backbone dihedrals. Related to this is the presence of native C2′-endo puckers in several nucleotides from or near those suites, namely A_{1n}, A_{L2} and A_{L3}. These C2′-endo puckers were well-maintained in simulations with the notable exception of A_{L2} (part of the loop region) which, as mentioned in the main text always transitioned into C3'-endo with all FFs except AMOEBA (Figures S1 and S10). For the sake of completeness, we also note that the native C3′-endo puckers (all other nucleotides except those noted above) were universally well reproduced by all FFs.

All results are fully supported by MD simulations performed under experimental solvent conditions. Following the Referee's suggestion, we undertook a more detailed evaluation of the influence of salt type and concentration in our simulations. Specifically, our benchmark study was originally conducted using a standard excess-salt concentration of 0.15 M KCl, whereas the crystallographic study[2] from which our starting structure was derived used 0.10 M NaCl and 0.020 M $MgCl_2$ (see the main text). To examine potential influences on the simulation results, we carried out an additional set of simulations using the OL3(SPC/E) FF combination. In response to another suggestion by the referee, these simulations were performed both at the standard simulation temperature of 298 K and at 100 K, which corresponds to the temperature used for crystallographic data collection.

Based on our analyses, we conclude that the salt conditions did not significantly affect the stability of SI and A-minor 0 interactions. Likewise, the balance between native and non-native SI interactions, the distribution of *syn* and *anti* N-glycosidic dihedral angles, and the stacking partners of A_{L2} were also unaffected. The occupancies of the 4BPh, sugar-phosphate, and sugar-based interactions were only slightly higher (Tables S6, S8 and S9). In contrast, the simulations performed at 100 K revealed no significant dynamical changes within the simulation timescale. In other words, all the interactions present in the native structure (Table S1) remained throughout the simulations with very little dynamics. We suggest this to be an expected result as at 100 K, the solute and the water box are essentially frozen, with all the thermal motions dampened and energy barriers for most conformational transitions insurmountable on our

simulation timescale. Note that the $AM0_B$ heavy atom distance increased in all 100 K simulations just after equilibration slightly above our 4 Å cutoff distance (see Methods in the main text). It subsequently never returns below this value on the simulation timescale due to lack of thermal motions and high friction of the water molecules surrounding the area.

Simulations of the non-truncated Kt-7 behave similarly to the truncated structure. The starting structure used for nearly all of our simulations (PDB ID: 4C40; see also main text) contains an asymmetric unit composed of a continuous strand incorporating both the longer and shorter strands of the kink-turn. When expanded to the biological unit via symmetry operations, two identical Kt-7 motifs are obtained, joined through their non-canonical stems (Figure S11). For the purpose of our benchmark simulations, only one of the Kt-7 motifs was used (see the main text). To confirm that this did not affect the results, we performed a set of control simulations using the full, non-truncated biological unit containing the co-joined Kt-7s, employing the OL3(SPC/E) FF combination. We observed that all the interactions were populated very similarly as in the truncated structure. We suggest that for all practical purposes, the results obtained from simulations of the truncated and non-truncated structures should be equivalent, including the relative performance of the individual RNA FFs.

Block averaging analysis of the convergence. To assess the adequacy of sampling, we performed block averaging of the RMSD variance of Kt-7, excluding the terminal and bulge residues. For additive FFs, the block size was increased from 5 to 5000 frames in steps of 5, and for polarizable FFs from 2 to 1000 frames in steps of 2. Plateau formation, indicative of structural convergence, was strongly dependent on the FF. For AMOEBA, four replicates reached or nearly reached the variance plateau, except for the replicate in which the A-minor I interaction formed. Among the additive FFs, ROC and OL3(SPC/E) showed the most converged results, with plateau formation in two replicates each. Overall, none of the FF exhibited a stable RMSD variance plateau across all replicates, indicating incomplete convergence by this criterion (Figure S12). Nevertheless, we still propose that the simulations were sufficiently long to evaluate the performance of each FF due to the strong qualitative agreement between the independent trajectories. While the individual trajectories are clearly not ergodic on the simulated timescales, the dominant dynamical features relevant for FF assessment of a fully kinked kink-turn were sufficiently captured.

Summary of recurrent FF issues observed in simulations of Kt-7 and tentative suggestions for refinement. We note that due to its unique structural characteristics, multitude of tertiary interactions, and availability of only static averaged X-ray structures, Kt-7 should be primarily used as a testing system during RNA FF refinements. In other words, after training the FF on other datasets, Kt-7 serves as a valuable benchmark for verifying whether the generalizability of the new parameters was not lost and to identify potential performance side-effects. Directly optimizing the FF parameters to achieve good performance on Kt-7 is in our opinion unlikely to

yield a generally applicable model. With that said, several recurrent issues were observed in our simulations that could serve as targets for future parametrizations.

The first concerns the balance between the populations of native and non-native SI interactions, which we observed with all FFs except the polarizable AMOEBA. We therefore suspect that the non-native SI may be an artifact arising from the absence of polarization. As such, it could be difficult to fully resolve in context of non-polarizable FFs. Nevertheless, this artifact was essentially mitigated by modification of the dihedral torsions in the ROC FF, in which the native SI population was substantially increased compared to all the other non-polarizable FFs. The second recurrent issue was the underrepresentation of non-canonical α/γ backbone conformations, the g^+/t and g^+/g^- suites. Although these backbone conformations are highly conserved for specific suites of the kink-turn motif, they are entirely lost with many of the FFs. Even in the best cases, a substantial canonical population is still introduced alongside the native non-canonical population, indicating that the FFs are generally over-parameterized for canonical RNA structures. Further refinement of the α/γ dihedral parameters to make them less biased towards canonical values, or of the coupled ε and ζ parameters, could potentially improve the description of Kt-7. However, such modifications of α/γ dihedral parameters are far from being trivial, since they can adversely affect other RNA backbone conformations; note that, after many years, the α/γ potential was recently refined in the OL21 DNA FF.[6] Last, we almost universally observed a loss of the *syn* conformation of the A_{L2} nucleotide, accompanied by a transition to the *anti*, which could indicate an *anti*-bias in some of the FFs and a potential for N-glycosidic χ dihedral parameters refinement. However, this also would not be a trivial task and considerably more experimental data supporting such a change would be needed.

In conclusion, we reiterate that Kt-7 is a system intended for testing transferability of RNA FFs rather than a system directly utilizable in parametrizations. This is for example demonstrated for the DESRES and CHARMM36 FFs, which suffer from visible and swift imbalances in simulations of Kt-7.

Supporting Information Tables

Table S1: **The RMSD (in Å), the H-bonding distances (in Å) of the interactions stabilizing the Kt-7, and the χ dihedral angle of A_{L2} (in °), as observed in different X-ray structures in the PDB database.**

Structures	RMSD[a]	SI	$AM0_A$	$AM0_B$	4BPh	Sugar-Phosphate	Sugar-Base	A_{L2}-χ
**4C40**	X	2.47	2.52	3.87	2.85	2.48	2.83	59.72
**4CS1**	0.103	2.53	2.46	3.98	2.88	2.39	2.80	59.72
**5FJ0**	0.449	2.64	2.58	3.80	3.09	2.80	2.66	54.34
**5FJ1**	0.355	2.63	2.52	3.88	2.95	2.59	2.77	54.22
**7EFG**	0.179	2.48	2.58	3.77	2.98	2.57	2.80	59.83
**7EFH**	0.43	2.70	2.61	2.98	2.92	3.03	2.84	64.91

[a]For each of the structures, a heavy-atom alignment to the 4C40 structure was performed prior to the RMSD calculation. Only the simulated part of the motif (see the main text Figure 1) was considered for the calculation.

Table S2: **Backbone dihedral angles and sugar pucker (in °) observed in the Kt-7 molecule excised from its X-ray structure (PDB: 4C40) and used as the starting structure in MD simulations.**[a]

Residue / Dihedral	α	β	γ	δ	ε	ζ	Sugar pucker[7]
G_{5n}	-	-	55.6	76.2	-144.0	-83.3	15.1
G_{4n}	-50.0	163.6	54.5	79.4	-142.0	-59.7	17.2
G_{3n}	-56.9	172.8	54.2	84.8	-136.0	-120.0	20.3
G_{2n}	**139.1**	-120.0	**177.4**	148.9	-110.2	-175.8	167.5
A_{1n}	-66.8	163.8	32.6	149.6	-85.2	101.2	162.9
G_{-1n}	**82.7**	-108.0	**-179.2**	84.1	-133.1	-87.9	3.2
C_{-2n}	-37.8	160.4	42.5	81.6	-147.1	-74.6	18.9
C_{-3n}	-63.2	173.8	56.4	79.9	-	-	21.3
G_{-3b}	-	-	82.8	85.4	-147.7	-72.7	10.7
G_{-2b}	-76.2	-177.1	58.8	78.4	-145.0	-63.9	11.0
C_{-1b}	-68.3	179.9	59.0	76.7	-145.8	-62.4	23.6
G_{L1}	-64.2	173.9	50.8	73.8	-126.8	-132.4	21.4
A_{L2}	**46.5**	-179.5	**-73.7**	153.1	-127.7	80.5	165.4
A_{L3}	**74.3**	-174.6	**59.7**	150.0	-86.8	-41.4	174.2
G_{1b}	-82.2	-151.0	68.6	153.3	-136.8	146.1	167.0
A_{2b}	-71.8	174.0	42.0	90.4	-136.9	-73.1	0.4
A_{3b}	-70.1	164.9	65.4	80.6	-132.4	-62.5	19.4
C_{4b}	-65.7	170.1	52.4	80.8	-148.9	-74.2	12.1
C_{5b}	-56.4	-178.0	43.6	78.7	-	-	13.3

[a]The combinations of α and γ values deviating from the canonical A-RNA backbone conformation are in bold. Note that the suite G_{3n}/G_{2n} was not included in the FF comparison because the values of this suite are unique to the 4C40 structure; the other Kt-7 structures always possesses canonical A-RNA values. In all our simulations, the suite immediately transitioned to A-RNA canonical values.

Table S3: **Overview of the methodological details of individual MD simulations.**[a]

FF label	MD software used	Periodic Box	Thermostat
Non-polarizable force fields[b]			
OL3	AMBER20	Cubic	Langevin
OL3-CP	AMBER20	Cubic	Langevin
OL3-HR2.7	AMBER20	Cubic	Langevin
PAK	AMBER20	Cubic	Langevin
ROC	AMBER20	Cubic	Langevin
Chen&Garcia	AMBER20	Cubic	Langevin
DESRES	AMBER20	Cubic	Langevin
DESAMBER	Gromacs2020	Rhombic dodecahedral	Stochastic velocity rescale
BSFF1	Gromacs2020	Rhombic dodecahedral	Stochastic velocity rescale
CHARMM36	Gromacs2020	Rhombic dodecahedral	Stochastic velocity rescale
Polarizable force fields			
CHARMM-Drude[c]	OpenMM 8.0	Cubic	Dual Langevin
AMOEBA[d]	Tinker-HP 1.3	Cubic	Stochastic velocity rescale

[a]The Monte Carlo barostat was used during the constant-pressure phases of all simulations.

[b]An integration step of 4 fs was used along with the leap-frog integrator.

[c]An integration time step of 1 fs was used with the Drude Langevin integrator.

[d]An integration time step of 2 fs was used with the RESPA integrator.

Table S4: **Irreversible unkinking of Kt-7 observed in MD simulations.**

FF label	Unkinking (μs)[a]
**$OL3_{OBPh,CP}$-gHBfix21**	R2(3.85)[b,c]
**$OL3_{R2.7}$**	R4(3.44)[b]
**ROC**	R2(8.48)
**DESRES**	R1(0.70)[b,c],R3(9.24),R5(6.71)[b,c]
**DES-Amber**	R4(6.96)[b,c]
**CHARMM36**	R1,R2,R3,R4,R5[b](all shortly after start)
**$CHARMM_{Drude}$**	R1(0.77)[b]

[a]Indicates the simulation time (μs) and replicate (R) in which the unkinking occurred. The RNA FFs not stated (see the main text Table 1) showed no instances of unkinking.
[b]Disruption of the first AG base pair (A_{1n}-G_{1b}).
[c]Disruption of the first AG base pair (A_{1n}-G_{1b}), followed by formation of a non-native AG base pair between A_{1n} and G_{L1}.

Table S5: **Irreversible disruptions of Kt-7's stems observed in MD simulations.**

FF label	Disruption of H-bonds (μs)[a]
**$OL3_{R2.7}$**	R4(4.11)
**DESRES**	R1(4.49),R4(5.23)
**BSSF1**	R1(2.25)
**CHARMM36**	R1(1.30),R2(0.17),R3(0.41),R4(0.27),R5(5.86)

[a]Indicates the replicate and simulation time (μs) at which a subsequently irreversible disruption of the H-bonds stabilizing the stem or stems first appeared.

Table S6: **Population analyses (in %) of the characteristic H-bond interactions forming the A-minor 0 and A-minor I interactions.**[a]

FF label / Interaction	$AM0_A$	$AM0_B$	AMI_A	AMI_B	AMI_C
**OL3(OPC)**	94.0 (± 3.2)	75.4 (± 10.4)	0.2 (± 0.4)	12.0 (± 21.6)	0.0 (± 0.0)
**OL3(SPC/E)**	97.8 (± 1.6)	74.2 (± 4.5)	0.0 (± 0.0)	1.2 (± 1.5)	0.0 (± 0.0)
**OL3(SPC/E)-1M**[b]	99.6 (± 0.5)	72.8 (± 4.7)	0.0 (± 0.0)	1.6 (± 1.4)	0.0 (± 0.0)
**OL3(SPC/E)-$MgCl_2$-NaCl-298K**[c]	97.2 (± 3.7)	70.8 (± 4.4)	0.0 (± 0.0)	2.0 (± 1.3)	0.0 (± 0.0)
**OL3(SPC/E)-$MgCl_2$-NaCl-100K**[c,d]	100.0 (± 0.0)	0.0 (± 0.0)	0.0 (± 0.0)	0.0 (± 0.0)	0.0 (± 0.0)
**OL3(SPC/E)-Non Truncated**[e]	99.0 (± 1.3)	79.3 (± 10.8)	0.0 (± 0.0)	2.7 (± 1.7)	0.0 (± 0.0)
**OL3(SPC/E)-L7Ae**	100.0 (± 0.0)	35.7 (± 2.4)	0.0 (± 0.0)	0.7 (± 0.5)	0.0 (± 0.0)
**$OL3_{R2.7}$**	85.0 (± 5.9)	36.3 (± 2.5)	0.0 (± 0.0)	5.3 (± 3.9)	0.0 (± 0.0)
**$OL3_{0BPh,CP}$-gHBfix21**	48.0 (± 7.9)	30.4 (± 7.7)	27.6 (± 11.3)	4.8 (± 2.7)	22.2 (± 8.1)
**PAK**	93.4 (± 7.7)	49.0 (± 4.9)	0.2 (± 0.4)	1.8 (± 1.0)	0.2 (± 0.4)
**ROC**	89.2 (± 19.7)	17.2 (± 4.8)	12.2 (± 19.4)	9.2 (± 13.6)	10.0 (± 19.5)
**Chen&Garcia**	50.2 (± 32.1)	14.2 (± 11.3)	40.4 (± 34.1)	35.2 (± 29.6)	39.6 (± 33.9)
**DESRES**	49.6 (± 9.9)	32.0 (± 11.4)	20.2 (± 14.3)	8.2 (± 9.7)	1.8 (± 1.5)
**DES-Amber**	64.8 (± 8.9)	50.2 (± 15.0)	4.8 (± 8.1)	10.2 (± 10.3)	1.8 (± 2.2)
**BSSF1**	74.8 (± 30.6)	64.6 (± 21.5)	1.2 (± 1.6)	15.4 (± 23.2)	0.4 (± 0.8)
**CHARMM36**	0.0 (± 0.0)	0.0 (± 0.0)	0.0 (± 0.0)	0.0 (± 0.0)	0.0 (± 0.0)
**$CHARMM_{Drude}$**	19.0 (± 11.7)	1.2 (± 1.6)	16.6 (± 7.9)	9.2 (± 17.9)	0.4 (± 0.8)
**AMOEBA**	90.4 (± 17.7)	19.2 (± 4.8)	9.0 (± 18.0)	1.2 (± 1.9)	8.2 (± 16.4)
**AMOEBA-L7Ae**	100.0 (± 0.0)	22.7 (± 3.1)	0.0 (± 0.0)	0.0 (± 0.0)	0.0 (± 0.0)

[a]The values refer to combined full simulation ensembles of the individual FFs. The standard deviation values refer to the variability among the individual replicates that make up the combined ensembles.

[b]A significantly increased KCl concentration of 1 M was used.

[c]The salt conditions used during the crystallization of the 4C40 structure (0.10 M NaCl and 0.020 M $MgCl_2$) were used.

[d]The temperature of 100 K utilized during the X-ray measurement of the 4C40 structure was used in the MD simulations.

[e]Simulation of a non-truncated structure (PDB: 4C40), containing two co-joined Kt-7 motifs. Data represent the mean percentage ± standard deviation from ten measurements (two Kt–7s × five replicates).

Table S7A: **Population analyses (in %) of the base pairing H-bonds in the AG base pairs of Kt-7.**[a]

FF Label / H-bond	A_{1n}(N6)-G_{1b}(N3)	G_{1b}(N2)-A_{1n}(N7)	A_{2b}(N6)-G_{2n}(N3)	G_{2n}(N2)-A_{2b}(N7)	A_{3b}(N6)-G_{3n}(N3)	G_{3n}(N2)-A_{3b}(N7)
**OL3(OPC)**	83.8 (± 31.4)	84.2 (± 31.6)	99.6 (± 0.5)	100.0 (± 0.0)	96.0 (± 1.8)	100.0 (± 0.0)
**OL3(SPC/E)**	100.0 (± 0.0)	100.0 (± 0.0)	100.0 (± 0.0)	100.0 (± 0.0)	96.6 (± 0.5)	100.0 (± 0.0)
**OL3(SPC/E)-1M**[b]	100.0 (± 0.0)	100.0 (± 0.0)	100.0 (± 0.0)	100.0 (± 0.0)	96.8 (± 0.4)	100.0 (± 0.0)
**OL3(SPC/E)-$MgCl_2$-NaCl-298K**[c]	99.8 (± 0.4)	99.8 (± 0.4)	100.0 (± 0.0)	100.0 (± 0.0)	98.0 (± 0.6)	100.0 (± 0.0)
**OL3(SPC/E)-$MgCl_2$-NaCl-100K**[c,d]	100.0 (± 0.0)	100.0 (± 0.0)	100.0 (± 0.0)	100.0 (± 0.0)	100.0 (± 0.0)	100.0 (± 0.0)
**OL3(SPC/E)-Non Truncated**[e]	100.0 (± 0.0)	100.0 (± 0.0)	99.9 (± 0.3)	100.0 (± 0.0)	95.9 (± 0.9)	100.0 (± 0.0)
**OL3(SPC/E)-L7Ae**	100.0 (± 0.0)	100.0 (± 0.0)	100.0 (± 0.0)	100.0 (± 0.0)	99.3 (± 0.5)	100.0 (± 0.0)
**$OL3_{R2.7}$**	100.0 (± 0.0)	100.0 (± 0.0)	100.0 (± 0.0)	100.0 (± 0.0)	99.3 (± 0.5)	100.0 (± 0.0)
**$OL3_{0BPh,CP}$-gHBfix21**	87.6 (± 22.3)	88.6 (± 21.3)	82.8 (± 9.4)	100.0 (± 0.0)	92.2 (± 0.7)	99.2 (± 0.4)
**PAK**	99.6 (± 0.5)	99.6 (± 0.5)	99.4 (± 0.5)	100.0 (± 0.0)	99.0 (± 0.9)	100.0 (± 0.0)
**ROC**	95.0 (± 10.0)	95.0 (± 10.0)	99.0 (± 1.3)	100.0 (± 0.0)	99.6 (± 0.5)	100.0 (± 0.0)
**Chen&Garcia**	76.6 (± 23.5)	81.2 (± 18.5)	84.6 (± 21.7)	100.0 (± 0.0)	99.4 (± 0.8)	99.8 (± 0.4)
**DESRES**	67.8 (± 30.9)	70.6 (± 31.1)	76.2 (± 26.7)	100.0 (± 0.0)	72.0 (± 23.8)	78.4 (± 26.2)
**DES-Amber**	93.0 (± 9.0)	95.0 (± 9.5)	99.0 (± 0.9)	100.0 (± 0.0)	95.8 (± 1.2)	99.6 (± 0.5)
**BSSF1**	63.4 (± 42.6)	63.6 (± 42.8)	84.2 (± 31.1)	100.0 (± 0.0)	80.8 (± 26.1)	86.2 (± 26.6)
**CHARMM36**	37.2 (± 37.9)	38.4 (± 39.3)	14.2 (± 16.3)	100.0 (± 0.0)	44.6 (± 39.1)	50.2 (± 36.0)
**$CHARMM_{Drude}$**	72.6 (± 34.4)	63.4 (± 36.5)	23.6 (± 13.8)	100.0 (± 0.0)	99.4 (± 0.5)	100.0 (± 0.0)
**AMOEBA**	99.8 (± 0.4)	100.0 (± 0.0)	86.2 (± 18.4)	100.0 (± 0.0)	98.4 (± 0.5)	98.0 (± 0.9)
**AMOEBA-L7Ae**	100.0 (± 0.0)	100.0 (± 0.0)	100.0 (± 0.0)	100.0 (± 0.0)	97.7 (± 0.9)	94.3 (± 5.2)

[a]The values refer to combined full simulation ensembles of the individual FFs. The standard deviation values refer to the variability among the individual replicates that make up the combined ensembles.

[b]A significantly increased KCl concentration of 1 M was used.

[c]The salt conditions used during the crystallization of the 4C40 structure (0.10 M NaCl and 0.020 M $MgCl_2$) were used.

[d]The temperature of 100 K utilized during the X-ray measurement of the 4C40 structure was used in the MD simulations.

[e]Simulation of a non-truncated structure (PDB: 4C40), containing two co-joined Kt-7 motifs. Data represent the mean percentage ± standard deviation from ten measurements (two Kt–7s × five replicates).

Table S7B: **Population analyses (in %) of the base pairing H-bonds in the canonical GC base pair of Kt-7.**[a]

FF	G_{-1n}(O6)-C_{-1b}(N4)	G_{-1n}(N1)-C_{-1b}(N3)	C_{-1b}(O2)-G_{-1n}(N2)
**OL3(OPC)**	100.0 (± 0.0)	100.0 (± 0.0)	100.0 (± 0.0)
**OL3(SPC/E)**	99.8 (± 0.4)	99.8 (± 0.4)	100.0 (± 0.0)
**OL3(SPC/E)-1M**[b]	99.6 (± 0.8)	99.6 (± 0.8)	99.6 (± 0.8)
**OL3(SPC/E)-$MgCl_2$-NaCl-298K**[c]	100.0 (± 0.0)	100.0 (± 0.0)	100.0 (± 0.0)
**OL3(SPC/E)-$MgCl_2$-NaCl-100K**[c,d]	100.0 (± 0.0)	100.0 (± 0.0)	100.0 (± 0.0)
**OL3(SPC/E)-Non Truncated**[e]	99.8 (± 0.4)	99.8 (± 0.4)	99.8 (± 0.4)
**OL3(SPC/E)-L7Ae**	100.0 (± 0.0)	100.0 (± 0.0)	100.0 (± 0.0)
**$OL3_{R2.7}$**	100.0 (± 0.0)	100.0 (± 0.0)	100.0 (± 0.0)
**$OL3_{0BPh,CP}$-gHBfix21**	100.0 (± 0.0)	100.0 (± 0.0)	100.0 (± 0.0)
**PAK**	100.0 (± 0.0)	100.0 (± 0.0)	100.0 (± 0.0)
**ROC**	100.0 (± 0.0)	100.0 (± 0.0)	100.0 (± 0.0)
**Chen&Garcia**	100.0 (± 0.0)	100.0 (± 0.0)	100.0 (± 0.0)
**DESRES**	100.0 (± 0.0)	100.0 (± 0.0)	100.0 (± 0.0)
**DES-Amber**	100.0 (± 0.0)	100.0 (± 0.0)	100.0 (± 0.0)
**BSSF1**	84.4 (± 30.2)	85.4 (± 28.2)	99.2 (± 1.6)
**CHARMM36**	57.8 (± 8.6)	43.6 (± 21.2)	50.8 (± 21.5)
**$CHARMM_{Drude}$**	100.0 (± 0.0)	100.0 (± 0.0)	100.0 (± 0.0)
**AMOEBA**	100.0 (± 0.0)	100.0 (± 0.0)	100.0 (± 0.0)
**AMOEBA-L7Ae**	100.0 (± 0.0)	100.0 (± 0.0)	100.0 (± 0.0)

[a]The values refer to combined full simulation ensembles of the individual FFs. The standard deviation values refer to the variability among the individual replicates that make up the combined ensembles.

[b]A significantly increased KCl concentration of 1 M was used.

[c]The salt conditions used during the crystallization of the 4C40 structure (0.10 M NaCl and 0.020 M $MgCl_2$) were used.

[d]The temperature of 100 K utilized during the X-ray measurement of the 4C40 structure was used in the MD simulations.

[e]Simulation of a non-truncated structure (PDB: 4C40), containing two co-joined Kt-7 motifs. Data represent the mean percentage ± standard deviation from ten measurements (two Kt–7s × five replicates).

Table S8: **Population analyses (in %) of the selected tertiary H-bonds of Kt-7, the N-glycosidic dihedral of A_{L2} and its stacking patterns.**[a]

FF label / Interaction	4BPh	Sugar-Phosphate	Sugar-Base	A_{L2}- *syn*	A_{L2}/A_{1n} stacking	A_{L2}/G_{L1} stacking
**OL3(OPC)**	2.2 (± 1.3)	33.6 (± 3.9)	69.2 (± 35.1)	8.0 (± 5.3)	19.6 (± 26.7)	56.6 (± 25.4)
**OL3(SPC/E)**	5.2 (± 3.0)	37.0 (± 3.8)	92.6 (± 4.5)	10.4 (± 9.3)	8.4 (± 5.3)	63.4 (± 6.5)
**OL3(SPC/E)-1M**[b]	6.0 (± 2.8)	42.2 (± 3.5)	99.0 (± 0.9)	17.0 (± 11.6)	6.8 (± 5.7)	65.0 (± 6.1)
**OL3(SPC/E)-$MgCl_2$-NaCl-298K**[c]	11.6 (± 2.1)	40.0 (± 4.8)	94.2 (± 8.2)	22.0 (± 3.8)	11.0 (± 3.0)	58.4 (± 2.1)
**OL3(SPC/E)-$MgCl_2$-NaCl-100K**[c,d]	100.0 (± 0.0)	100.0 (± 0.0)	100.0 (± 0.0)	100.0 (± 0.0)	96.6 (± 3.8)	0.0 (± 0.0)
**OL3(SPC/E)-Non Truncated**[e]	11.8 (± 8.7)	40.1 (± 4.3)	96.6 (± 6.0)	15.6 (± 20.8)	9.5 (± 11.3)	61.7 (± 12.5)
**OL3(SPC/E)-L7Ae**	20.0 (± 0.8)	100.0 (± 0.0)	100.0 (± 0.0)	100.0 (± 0.0)	84.3 (± 0.5)	0.0 (± 0.0)
**$OL3_{R2.7}$**	3.3 (± 0.5)	30.0 (± 5.4)	58.7 (± 12.5)	3.7 (± 0.5)	19.7 (± 4.0)	61.0 (± 5.7)
**$OL3_{0BPh,CP}$-gHBfix21**	7.0 (± 4.5)	8.8 (± 4.8)	23.6 (± 11.1)	3.0 (± 1.3)	19.8 (± 2.8)	53.4 (± 4.8)
**PAK**	18.4 (± 8.7)	38.0 (± 11.1)	84.8 (± 18.0)	27.6 (± 16.5)	20.8 (± 9.4)	57.2 (± 9.2)
**ROC**	8.8 (± 9.2)	13.2 (± 7.0)	93.8 (± 12.4)	2.2 (± 0.4)	2.0 (± 2.6)	67.0 (± 2.1)
**Chen&Garcia**	13.8 (± 6.4)	13.8 (± 8.5)	61.2 (± 20.4)	0.4 (± 0.5)	20.4 (± 22.7)	47.8 (± 17.6)
**DES-Amber**	1.4 (± 0.5)	39.6 (± 10.9)	14.0 (± 6.6)	2.6 (± 2.1)	28.0 (± 9.9)	48.6 (± 13.4)
**BSSF1**	3.0 (± 2.3)	33.8 (± 3.1)	64.2 (± 33.7)	9.2 (± 6.2)	17.6 (± 28.3)	61.6 (± 30.6)
**AMOEBA**	47.2 (± 9.5)	74.0 (± 6.0)	95.4 (± 8.2)	42.4 (± 29.8)	55.8 (± 24.8)	22.4 (± 27.6)
**AMOEBA-L7Ae**	27.3 (± 4.7)	99.0 (± 0.0)	99.7 (± 0.5)	100.0 (± 0.0)	89.0 (± 1.4)	0.0 (± 0.0)

[a]The values refer to combined full simulation ensembles of the individual FFs when the signature interaction was present. The standard deviation values refer to the variability among the individual replicates that make up the combined ensembles. Note that the A_{L2} nucleotide is involved in extensive crystal packing interactions (see the main text and Figure S1).

[b]A significantly increased KCl concentration of 1 M was used.

[c]The salt conditions used during the crystallization of the 4C40 structure (0.10 M NaCl and 0.020 M $MgCl_2$) were used.

[d]The temperature of 100 K utilized during the X-ray measurement of the 4C40 structure was used in the MD simulations.

[e]Simulation of a non-truncated structure (PDB: 4C40), containing two co-joined Kt-7 motifs. Data represent the mean percentage ± standard deviation from ten measurements (two Kt–7s × five replicates). Note that in one replicate, one of the kink-turns maintained the A_{L2}-syn conformation unusually long, but it eventually permanently transitioned into *anti* as in all the other replicates.

Table S9: **Population analyses (in %) of the signature interaction (SI).**[a]

FF label	SI
**OL3(OPC)**	90.6 (± 2.4)
**OL3(SPC/E)**	92.8 (± 1.2)
**OL3(SPC/E)-1M**[b]	93.8 (± 0.4)
**OL3(SPC/E)-$MgCl_2$-NaCl-298K**[c]	93.6 (± 1.5)
**OL3(SPC/E)-$MgCl_2$-NaCl-100K**[c,d]	100.0 (± 0.0)
**OL3(SPC/E)-Non Truncated**[e]	93.3 (± 2.1)
**OL3(SPC/E)-L7Ae**	100.0 (± 0.0)
**$OL3_{R2.7}$**	91.7 (± 1.7)
**$OL3_{0BPh,CP}$-gHBfix21**	92.4 (± 9.7)
**PAK**	91.8 (± 1.9)
**ROC**	97.4 (± 4.7)
**Chen&Garcia**	98.4 (± 0.8)
**DESRES**	73.6 (± 10.0)
**DES-Amber**	88.8 (± 3.5)
**BSSF1**	78.2 (± 28.8)
**CHARMM36**	10.0 (± 12.0)
**$CHARMM_{Drude}$**	30.8 (± 7.7)
**AMOEBA**	98.8 (± 1.5)
**AMOEBA-L7Ae**	100.0 (± 0.0)

[a]The values refer to combined average of simulation ensembles of the individual FFs. The standard deviation values (in parentheses) refer to the variability among the individual replicates that make up the combined ensembles. Both native and non-native SI conformations are included.

[b]A significantly increased KCl concentration of 1 M was used.

[c]The salt conditions used during the crystallization of the 4C40 structure (0.10 M NaCl and 0.020 M $MgCl_2$) were used.

[d]The temperature of 100 K utilized during the X-ray measurement of the 4C40 structure was used in the MD simulations.

[e]Simulation of a non-truncated structure (PDB: 4C40), containing two co-joined Kt-7 motifs. Data represent the mean percentage ± standard deviation from ten measurements (two Kt–7s × five replicates).

Table S10: **Population analyses (in %) of tertiary RNA-RNA interactions in L7Ae/Kt-7 protein-RNA complex simulations and their comparison with the free Kt-7.**[a]

Interaction / FF Label	OL3(SPC/E)	OL3(SPCE)-L7Ae	AMOEBA	AMOEBA-L7Ae
**SI**	92.8 (± 1.2)	100.0 (± 0.0)	98.6 (± 1.4)	100.0 (± 0.0)
**Native SI**	30.0 (± 6.0)	99.3 (± 0.5)	98.6 (± 1.4)	100.0 (± 0.0)
**Non-native SI**	63.0 (± 6.0)	0.0 (± 0.0)	0.0 (± 0.0)	0.0 (± 0.0)
**$AM0_A$**	97.8 (± 1.6)	100.0 (± 0.0)	90.2 (± 17.6)	100.0 (± 0.0)
**$AM0_B$**	74.2 (± 4.5)	35.7 (± 2.4)	18.2 (± 4.8)	22.7 (± 3.1)
**AMI_A**	0.0 (± 0.0)	0.0 (± 0.0)	9.0 (± 18.0)	0.0 (± 0.0)
**AMI_B**	1.2 (± 1.5)	0.7 (± 0.5)	1.4 (± 2.3)	0.0 (± 0.0)
**AMI_C**	0.0 (± 0.0)	0.0 (± 0.0)	0.0 (± 0.0)	0.0 (± 0.0)
syn	10.4 (± 9.3)	100.0 (± 0.0)	32.6 (± 24.8)	100.0 (± 0.0)
**A_{L2}/A_{1n} stacking**	8.4 (± 5.3)	84.3 (± 0.5)	52.6 (± 23.3)	89.0 (± 1.4)
**A_{L2}/G_{L1} stacking**	63.4 (± 6.5)	0.0 (± 0.0)	24.0 (± 26.2)	0.0 (± 0.0)
**4BPh**	5.2 (± 3.0)	20.0 (± 0.8)	46.0 (± 9.7)	27.3 (± 4.7)
**Sugar-Phosphate**	37.0 (± 3.8)	100.0 (± 0.0)	74.6 (± 5.9)	99.0 (± 0.0)
**Sugar-Base**	92.6 (± 4.5)	100.0 (± 0.0)	95.2 (± 8.6)	99.7 (± 0.5)

[a]The values refer to combined simulation ensembles of the individual FFs. The standard deviation values refer to the variability among the individual replicates that make up the combined ensembles.

Table S11: **Time intervals (in µs) during which the A-minor I interaction was present in the individual MD simulations.**[a]

FF label / Replicate	R1	R2	R3	R4	R5
OL3(SPC/E)-AMI	0-2.31	0-3.49	0-20	0-4.17	0-1.57;1.78-2.44
$OL3_{0BPh,CP}$-gHBfix21	2.07-4.32;4.83-5.84	0.94-1.62;2.64-3.85	6.84-7.61	0.58-3.24;9.77-9.88	5.45-5.74;7.50-10
Chen&Garcia	8.25-10	1.5-10	4.59-6.59;9.99-10	2.05-6.64;6.94-10	x
ROC	X	x	x	x	5.05-10
AMOEBA	X	x	x	1.37-2.5	x

[a]Replicates marked with "x" did not populate the A-minor I interaction. Note that the OL3(SPC/E)-AMI simulations were started from a structure with the A-minor I interaction already present (see the main text Methods). FFs not shown did not sample the A-minor I interaction.

Table S12: **Population analyses (in %) of the characteristic Kt-7 interactions whenever the A-minor I interaction was present.**[a]

Interaction / FF label	OL3(SPC/E)-AMI	Chen&Garcia	$OL3_{0BPh,CP}$-gHBfix21	ROC	AMOEBA
SI	99.0	100.0	98.0	99.0	100.0
Native SI	85.0	92.0	98.0	95.0	100.0
Non-native SI	13.8	7.0	0.0	4.0	0.0
syn	52.2	0.0	1.0	4.0	0.0
A_{L2}/A_{1n}	46.0	38.0	4.0	14.0	0.0
A_{L2}/G_{L1}	25.4	41.0	80.0	60.0	95.0
4BPh	79.2	17.0	26.0	51.0	77.0
Sugar-Phosphate	80.6	20.0	8.0	42.0	71.0
Sugar-Base	81.2	63.0	70.0	38.0	53.0

[a]The table shows population of the selected structural features during those parts of trajectories where the Kt-7 adopted the A-minor I conformation. Note that the OL3(SPC/E)-AMI simulations were started from a structure with the A-minor I interaction already present (see the main text Methods). For the remaining FFs, the A-minor I interaction was sometimes populated in simulations starting from the A-minor 0 conformation. The Table mainly illustrates the positive correlation between the native SI (see the main text Figure 3) and the A-minor I interaction.

Table S13: **Potassium binding-sites population analyses (in %).**[a]

OL3(OPC)		OL3(SPC/E)		$OL3_{0BPh,CP}$-gHBfix21		OL3(SPC/E)-1M		DES-Amber	
**Acceptor**	**Population**	**Acceptor**	**Population**	**Acceptor**	**Population**	**Acceptor**	**Population**	**Acceptor**	**Population**
G_{2n}(O6)	124.4	G_{2n}(O6)	150.3	G_{2n}(O6)	135.6	G_{3n}(O6)	116.4	G_{3n}(N7)	59.5
G_{3n}(O6)	103.4	G_{3n}(O6)	145.8	G_{3n}(O6)	132.0	G_{2n}(O6)	115.9	G_{2n}(O6)	58.8
G_{3n}(N7)	75.7	G_{3n}(N7)	84.3	G_{3n}(N7)	83.1	G_{3n}(N7)	66.9	G_{3n}(O6)	57.0
		G_{4n}(N7)	79.5	G_{4n}(OP2)	70.9	G_{4n}(N7)	66.4	**AMOEBA**	
		G_{4n}(OP2)	67.4			G_{5n}(O6)	58.2	**Acceptor**	**Population**
		G_{5n}(O6)	58.5			G_{4n}(OP2)	58.0	G_{3n}(O6)	90.4
		G_{4n}(O6)	58.1					G_{2n}(O6)	64.1
		G_{1b}(N7)	56.1					G_{4n}(OP2)	50.0
		G_{2n}(N7)	51.2					G_{3n}(N7)	50.0

$OL3_{R2.7}$		PAK		ROC		Chen&Garcia		BSFF1	
**Acceptor**	**Population**	**Acceptor**	**Population**	**Acceptor**	**Population**	**Acceptor**	**Population**	**Acceptor**	**Population**
G_{2n}(O6)	126.3	G_{2n}(O6)	121.9	G_{2n}(O6)	115.8	G_{3n}(O6)	87.1	G_{2n}(O6)	127.3
G_{3n}(O6)	107.4	G_{3n}(O6)	104.0	G_{3n}(O6)	100.6	G_{2n}(O6)	84.3	G_{3n}(O6)	122.3
G_{3n}(N7)	73.7	G_{3n}(N7)	78.9	G_{3n}(N7)	69.2	G_{3n}(N7)	74.8	G_{3n}(N7)	92.7
G_{4n}(OP2)	67.5	G_{4n}(OP2)	53.1	G_{4n}(OP2)	57.6	G_{4n}(N7)	53.6	G_{4n}(OP2)	77.1
G_{1b}(N7)	53.4	G_{1b}(N7)	52.1	G_{1b}(N7)	55.6			G_{4n}(N7)	61.5
				G_{4n}(N7)	52.6				

[a]Ion-binding sites with a population above 50% are listed. Populations of over 100% indicate more than one potassium ion being present at the site on average. This analysis was done over the combined simulation ensemble of all trajectories when the SI interaction was present.

Table S14: **Population analyses (in %) of the potassium binding-sites in L7Ae/Kt-7 protein-RNA complex simulations and their comparison with the free Kt-7.**[a]

OL3(SPC/E)		OL3(SPC/E)-L7Ae	
**Acceptor**	**Population**	**Acceptor**	**Population**
G_{2n}(O6)	150.3	G_{3n}(O6)	149.9
G_{3n}(O6)	145.8	A_{L3}(HO2')	95.4
G_{3n}(N7)	84.3	A_{L3}(O2')	95.3
G_{4n}(N7)	79.5	G_{2n}(O6)	75.1
G_{4n}(OP2)	67.4	G_{1b}(OP1)	71.6
G_{5n}(O6)	58.5	G_{4n}(N7)	71.0
G_{4n}(O6)	58.1	A_{L3}(O3')	69.0
G_{1b}(N7)	56.1	G_{3n}(N7)	67.7
G_{2n}(N7)	51.2	G_{5n}(O6)	52.3
		G_{4n}(O6)	50.7
**AMOEBA**		**AMOEBA-L7Ae**	
**Acceptor**	**Population**	**Acceptor**	**Population**
G_{3n}(O6)	90.4	G_{3n}(O6)	80.1
G_{2n}(O6)	64.1	G_{3n}(N7)	63.3
G_{4n}(OP2)	50.0	G_{2n}(O6)	62.2
G_{3n}(N7)	50.0	G_{4n}(OP2)	61.4

[a]Ion-binding sites with a population above 50% are listed. Populations of over 100% indicate more than one potassium ion being present at the site on average. The same ion-binding sites appearing with both FFs are color-coded for clarity.

Table S15: **Population analyses (in %) of the protein-RNA H-bonds formed in L7Ae/Kt-7 complex simulations.**[a]

OL3(SPC/E)		AMOEBA	
**H-bond (Donor - acceptor)**	**Population**	**H-bond (Donor - acceptor)**	**Population**
A_{L3}(OP2) - Thr51(N)	93.9	Glu53(OE) - G_{2n}(O2')	87.1
A_{L3}(OP1) - Ala112(N)	85.7	A_{L3}(OP2) - Thr51(OG1)	81.9
A_{L3}(OP2) - Thr51(OG1)	83.6	G_{3n}(OP2) - Lys56(NZ)	71.6
G_{1b}(O6) - Glu53(N)	67.5	A_{L3}(OP2) - Thr51(N)	58.3
Glu53(OE) - G_{2n}(O2')	55.7	G_{4n}(OP2) - Lys56(NZ)	57.5
G_{3n}(OP2) - Lys56(NZ)	43.2	A_{L3}(OP1) - Ala112(N)	52.8
Glu53(OE) - G_{1b}(N2)	38.7	G_{1b}(O6) - Glu53(N)	50.7
G_{3n}(OP1) - Arg60(NH2)	37.2	A_{L3}(N7) - Lys98(NZ)	47.2
		Glu53(OE) - G_{1b}(N1)	33.6
G_{2n}(N7) - Lys56(NZ)[b]	19.7	G_{2n}(N7) - Lys56(NZ)[b]	5.4

[a]Protein-RNA H-bonds with population above 30% are listed.

[b]The G_{2n}(N7) - Lys56(NZ) H-bond and generally the proximity of the lysine side-chain was suggested to cause a decrease in K^+ occupancy of the major G_{2n}(O6) K^+ binding site in OL3(SPC/E) FF (see above). Because of its potential importance, the H-bond is listed in the Table even though it does not meet the population cut-off.

Table S16: **Population analyses (in %) of the ion binding-sites of Kt-7 in MD simulations using different salt conditions.**[a]

OL3(SPC/E) - 150 mM KCl		OL3(SPC/E) - 100 mM + 20 $MgCl_2$ mM	
**Acceptor**	**Population(%)**	**Acceptor**	**Population(%)**
G_{2n}(O6)	150.3	**Na^+**	
G_{3n}(O6)	145.8	G_{2n}(O6)	89.8
G_{3n}(N7)	84.3	G_{3n}(O6)	76.1
G_{4n}(N7)	79.5	G_{3n}(N7)	44.9
G_{4n}(OP)	72.9	**Mg^{2+}**	
G_{5n}(O6)	58.5	G_{L1}(OP)	42.7
G_{4n}(O6)	58.1	A_{L2}(OP)	37.6
G_{1b}(N7)	56.1	G_{3n}(OP)	15.1
G_{2n}(N7)	51.2		

[a]Ion-binding sites of K^+ with a population above 50% are listed. Populations of over 100% indicate more than one potassium ion being present at the site on average. As the populations of Na^+ and Mg^{2+} are considerably lower, only the three most frequently visited ion-binding states are shown.

Supporting Information Figures

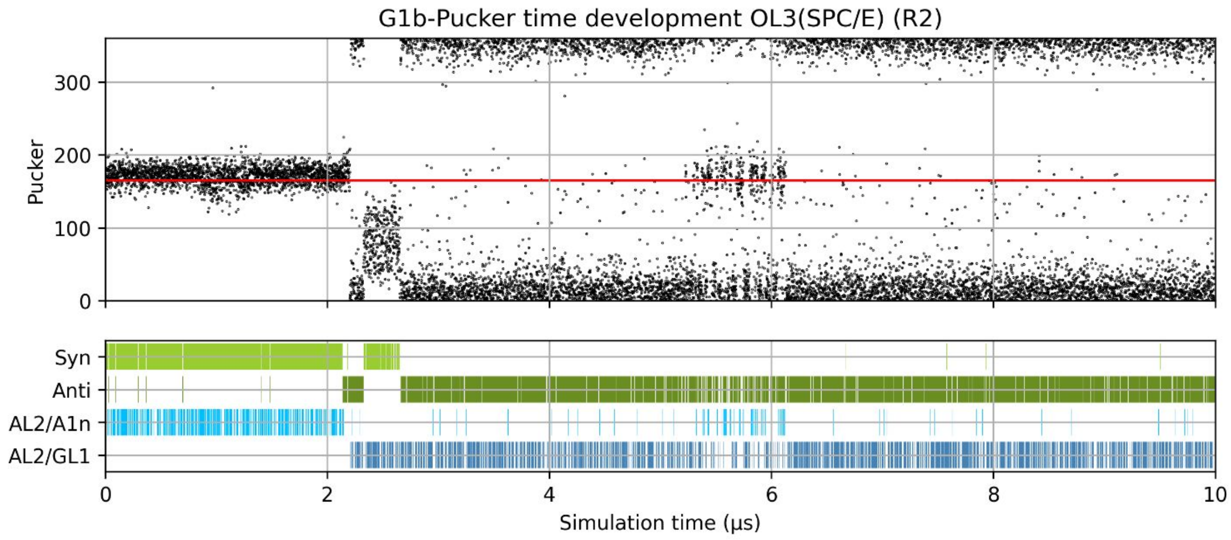

Figure S1A: **Time development of the A_{L2} sugar pucker in replicate 2 of the OL3(SPC/E) simulations.** The upper plot shows the pucker at each frame with the horizontal red line representing the experimental value (see Table S1). The bottom bar plot depicts presence of the N-glycosidic dihedral angle of A_{L2} in either the *syn* or the *anti* conformations using lighter and darker green respectively. The stacking pattern of A_{L2} (A_{L2}/A_{1n} or A_{L2}/G_{L1}) is then shown in lighter and darker blue respectively. Changes in A_{L2} sugar pucker are highly correlated with changes among these interactions. The other replicates show identical correlations.

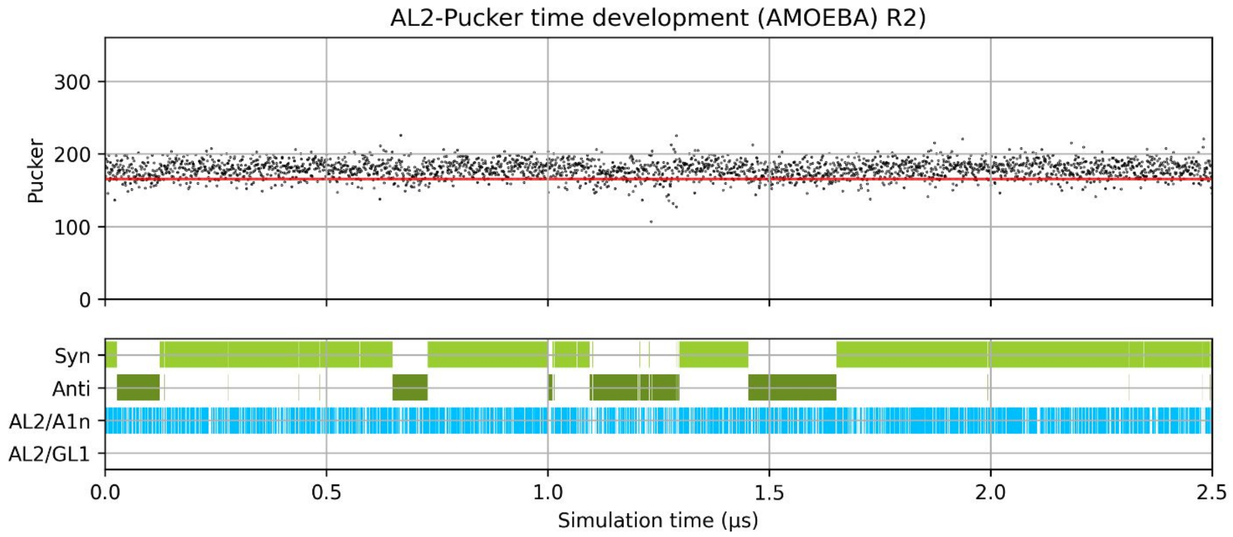

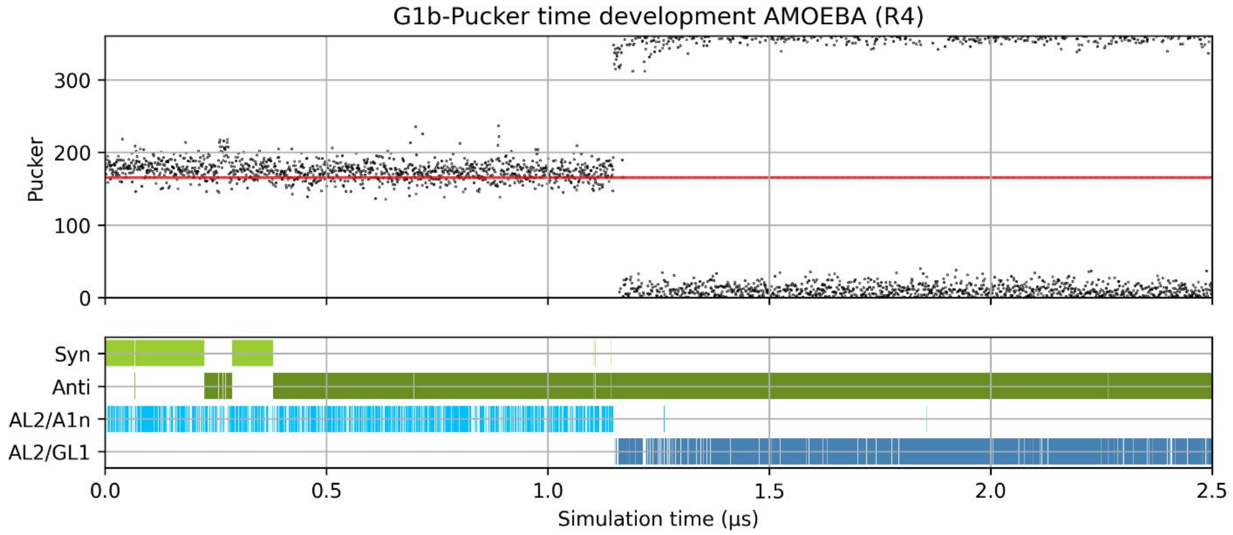

Figure S1B: **Time development of the A_{L2} sugar pucker in replicate 2 and 4 of the AMOEBA simulations.** The upper plot shows the pucker at each frame with the horizontal red line representing the experimental value (see Table S1). The bottom bar plot depicts presence of the N-glycosidic dihedral angle of A_{L2} in either the *syn* or the *anti* conformations using lighter and darker green respectively. The stacking pattern of A_{L2} (A_{L2}/A_{1n} or A_{L2}/G_{L1}) is then shown in lighter and darker blue respectively. Changes in A_{L2} sugar pucker are highly correlated with changes among these interactions. The other replicates show identical correlations.

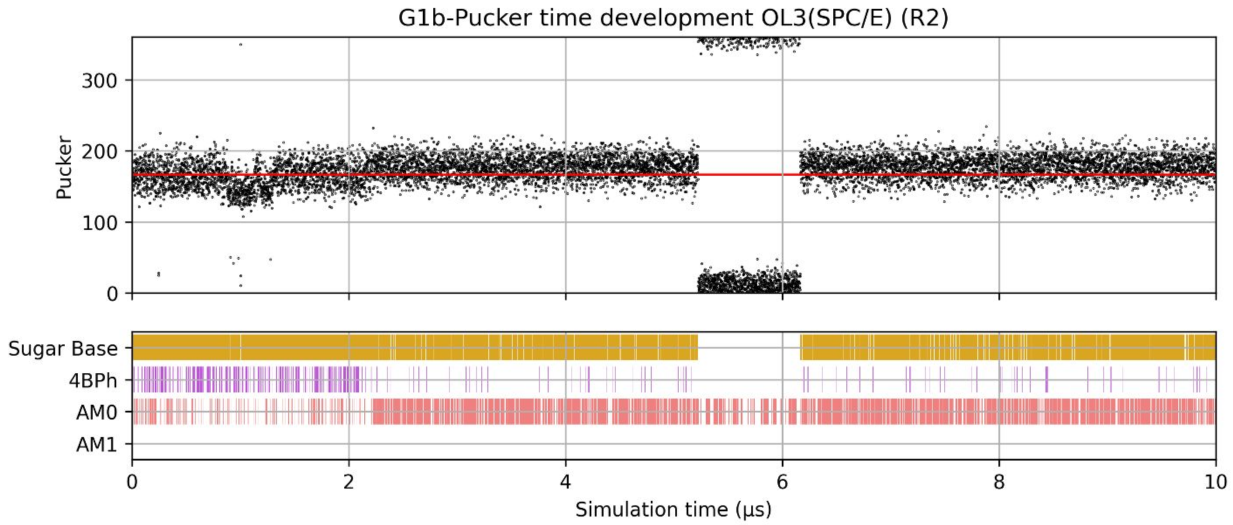

Figure S2A: **Time development of the G_{1b} sugar pucker in replicate 2 of the OL3(SPC/E) simulations.** The upper plot shows the pucker at each frame with the horizontal red line representing the experimental value (see Table S1). The bottom bar plot depicts presence of the sugar base (G_{1b}(O2')-G_{2n}(N2) and the 4BPh interaction (A_{2b}(OP2)-G_{3n}(N1/N2)) using yellow and purple respectively. Formation of A minor 0 or A minor 1 interaction is then shown in lighter and darker red respectively. Changes in G_{1b} sugar pucker are highly correlated with changes among these interactions. The other replicates show identical correlations.

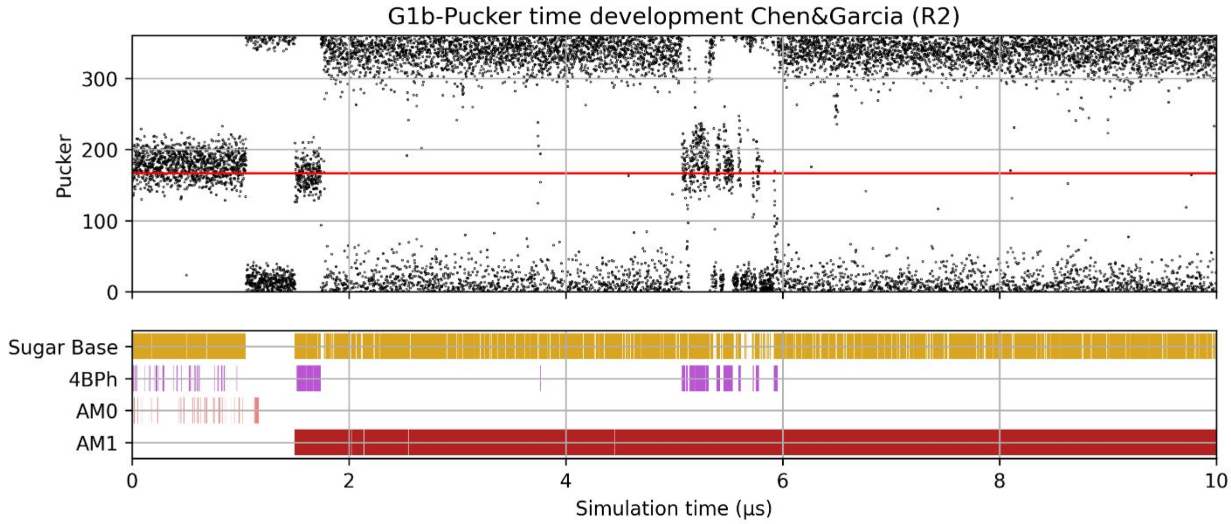

Figure S2B: **Time development of the G_{1b} sugar pucker in replicate 2 of the Chen&Garcia simulations.** The upper plot shows the pucker at each frame with the horizontal red line representing the experimental value (see Table S1). The bottom bar plot depicts presence of the sugar base (G_{1b}(O2')-G_{2n}(N2) and the 4BPh interaction (A_{2b}(OP2)-G_{3n}(N1/N2)) using yellow and purple respectively. Formation of A minor 0 or A minor 1 interaction is then shown in lighter and darker red respectively. Changes in G_{1b} sugar pucker are highly correlated with changes among these interactions. The other replicates show identical correlations.

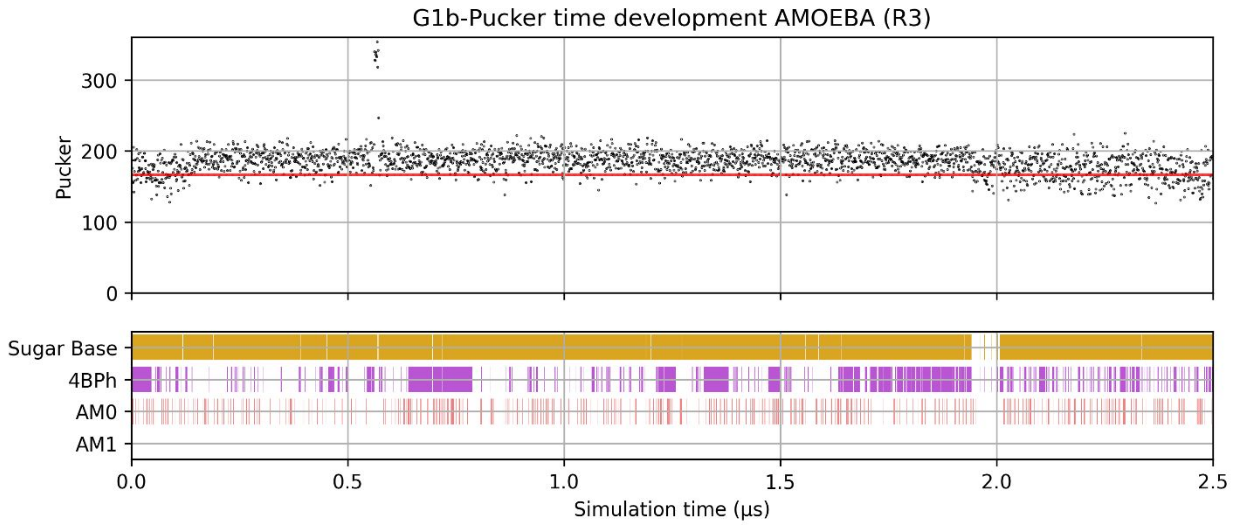

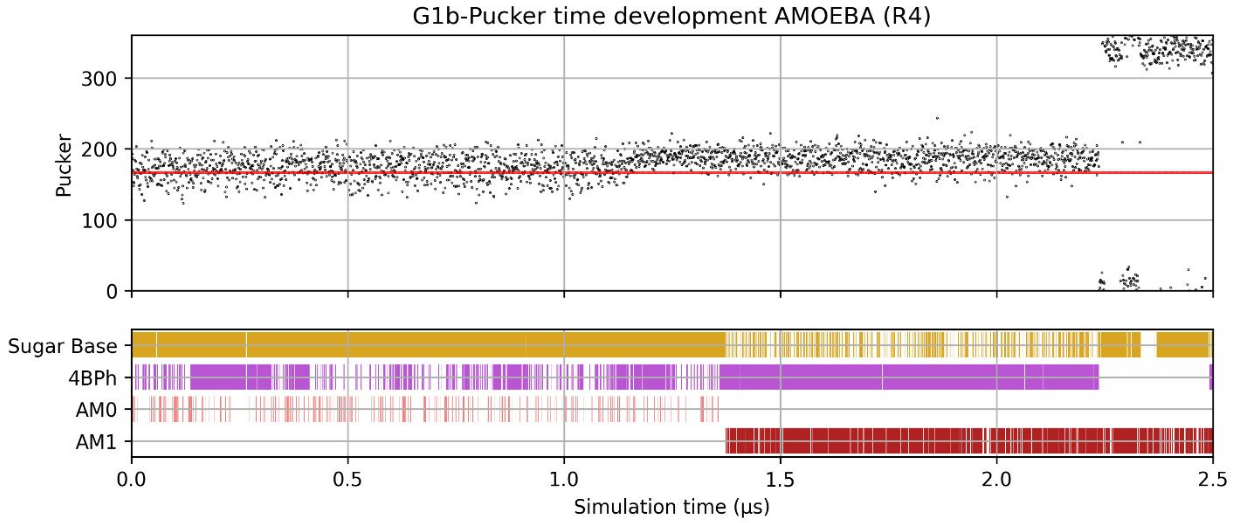

Figure S2C: **Time development of the G_{1b} sugar pucker in replicate 3 and 4 of the AMOEBA simulations.** The upper plot shows the pucker at each frame with the horizontal red line representing the experimental value (see Table S1). The bottom bar plot depicts presence of the sugar base (G_{1b}(O2')-G_{2n}(N2) and the 4BPh interaction (A_{2b}(OP2)-G_{3n}(N1/N2)) using yellow and purple respectively. Formation of A minor 0 or A minor 1 interaction is then shown in lighter and darker red respectively. Changes in G_{1b} sugar pucker are highly correlated with changes among these interactions. The other replicates show identical correlations.

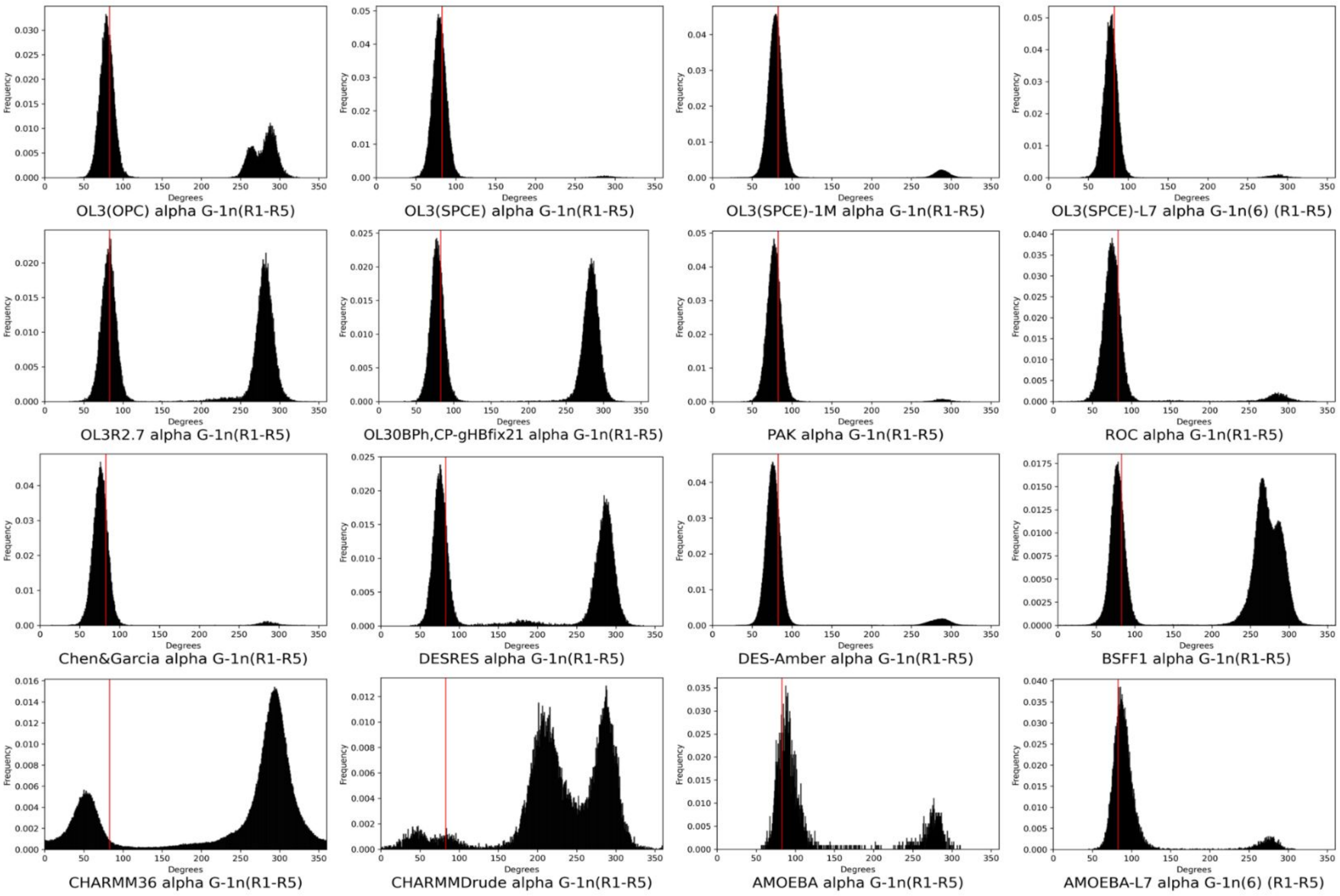

Figure S3A: **Histograms of the backbone dihedral α of suite A_{1n}/G_{-1n} for all tested FFs.** Values for combined simulation ensembles are shown. The vertical red line represents the experimental value (see Table S1).

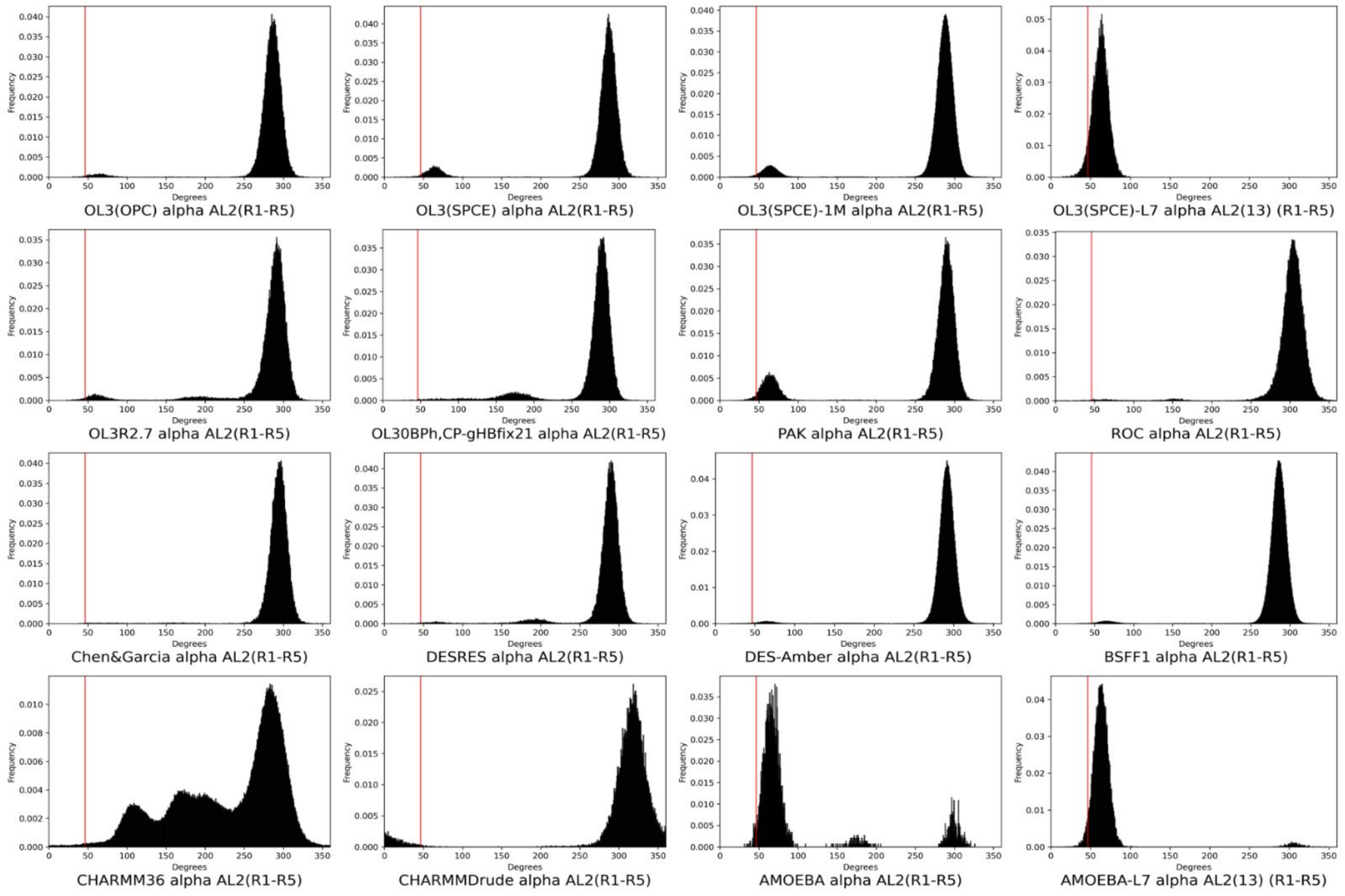

Figure S3B: **Histograms of the backbone dihedral α of suite G_{L1}/A_{L2} for all tested FFs.** Values for combined simulation ensembles are shown. The vertical red line represents the experimental value (see Table S1)

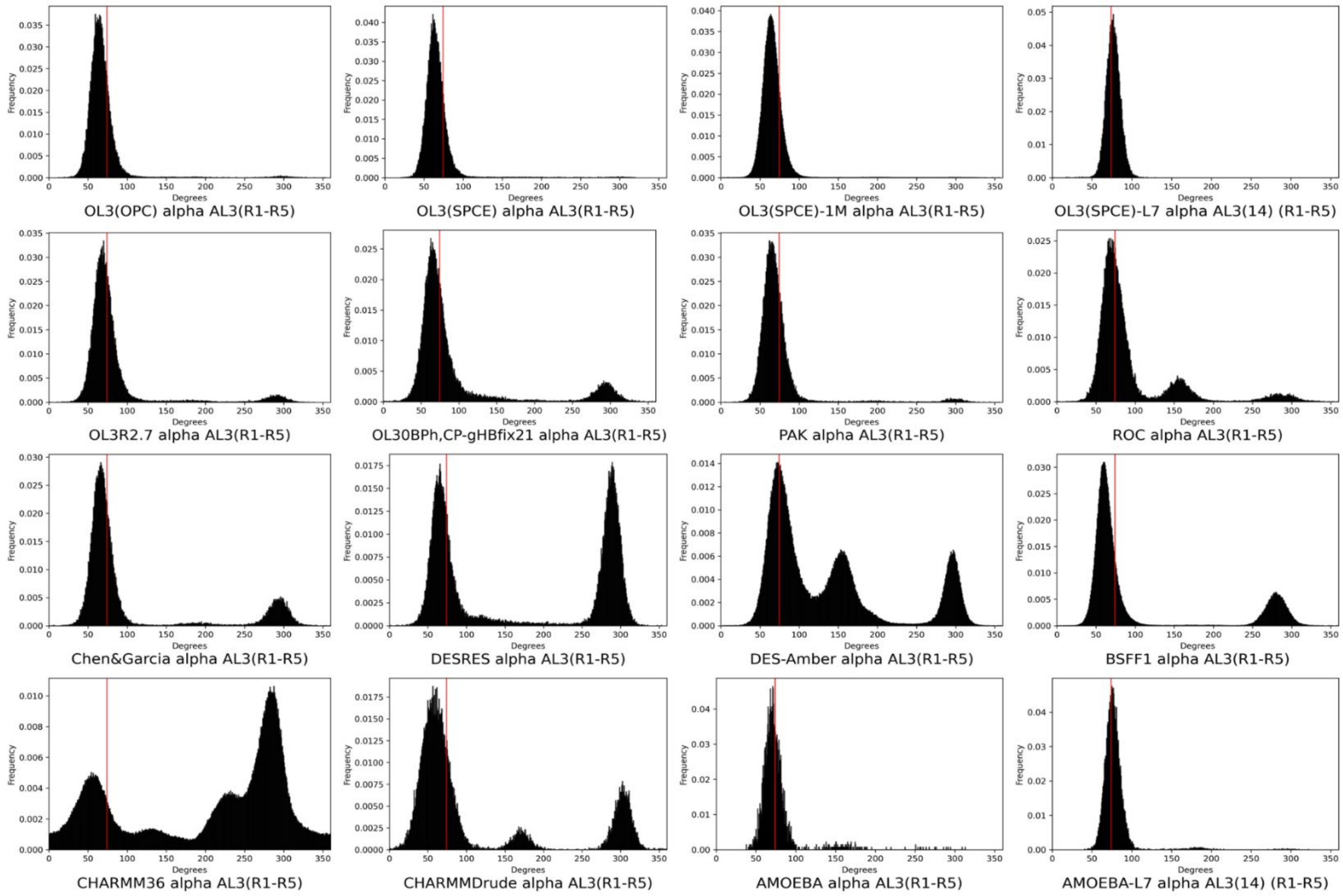

Figure S3C: **Histograms of the backbone dihedral α of suite A_{L2}/A_{L3} for all tested FFs.** Values for combined simulation ensembles are shown. The vertical red line represents the experimental value (see Table S1).

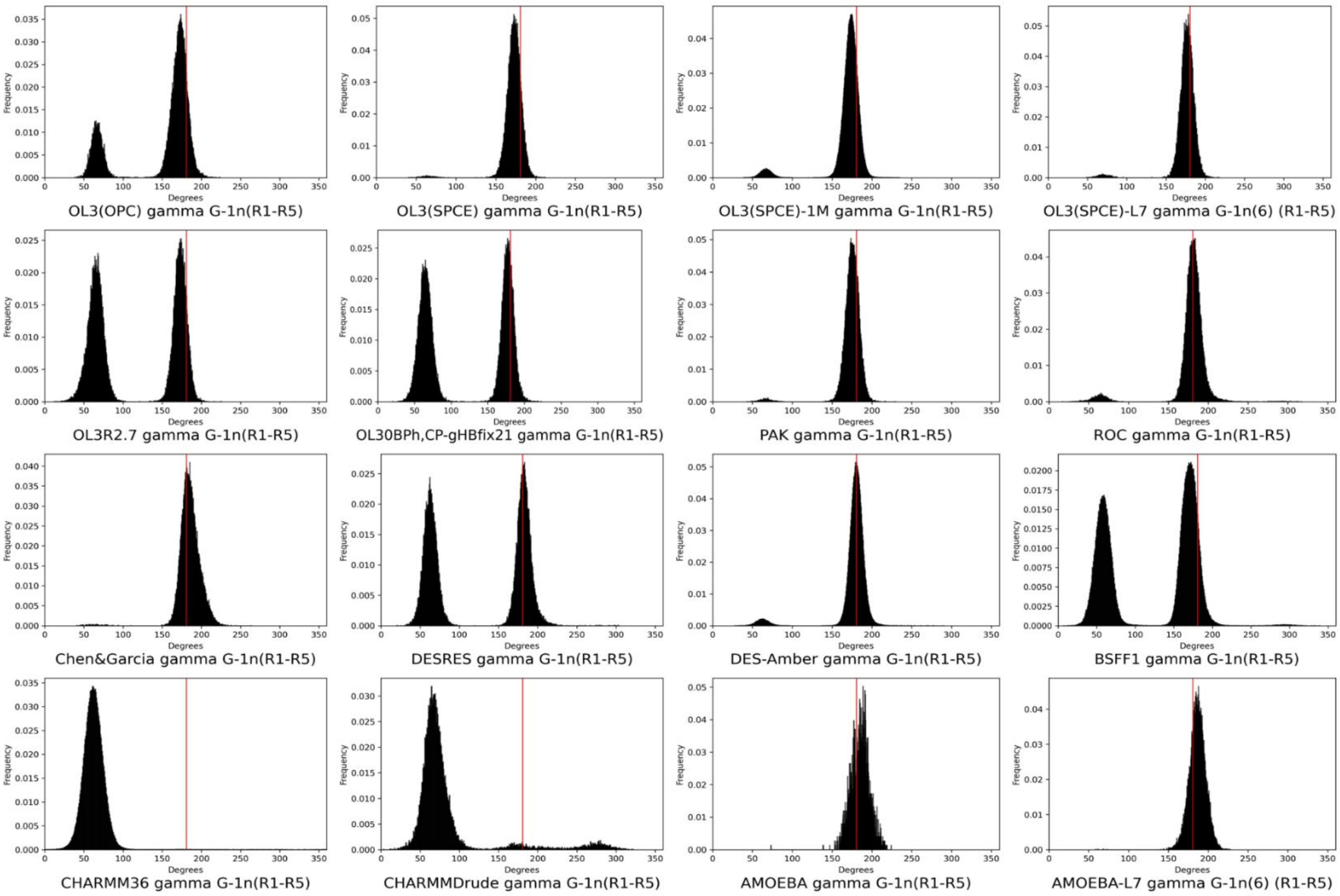

Figure S4A: **Histograms of the backbone dihedral γ of suite A_{1n}/G_{-1n} for all tested FFs.** Values for combined simulation ensembles are shown. The vertical red line represents the experimental value (see Table S1).

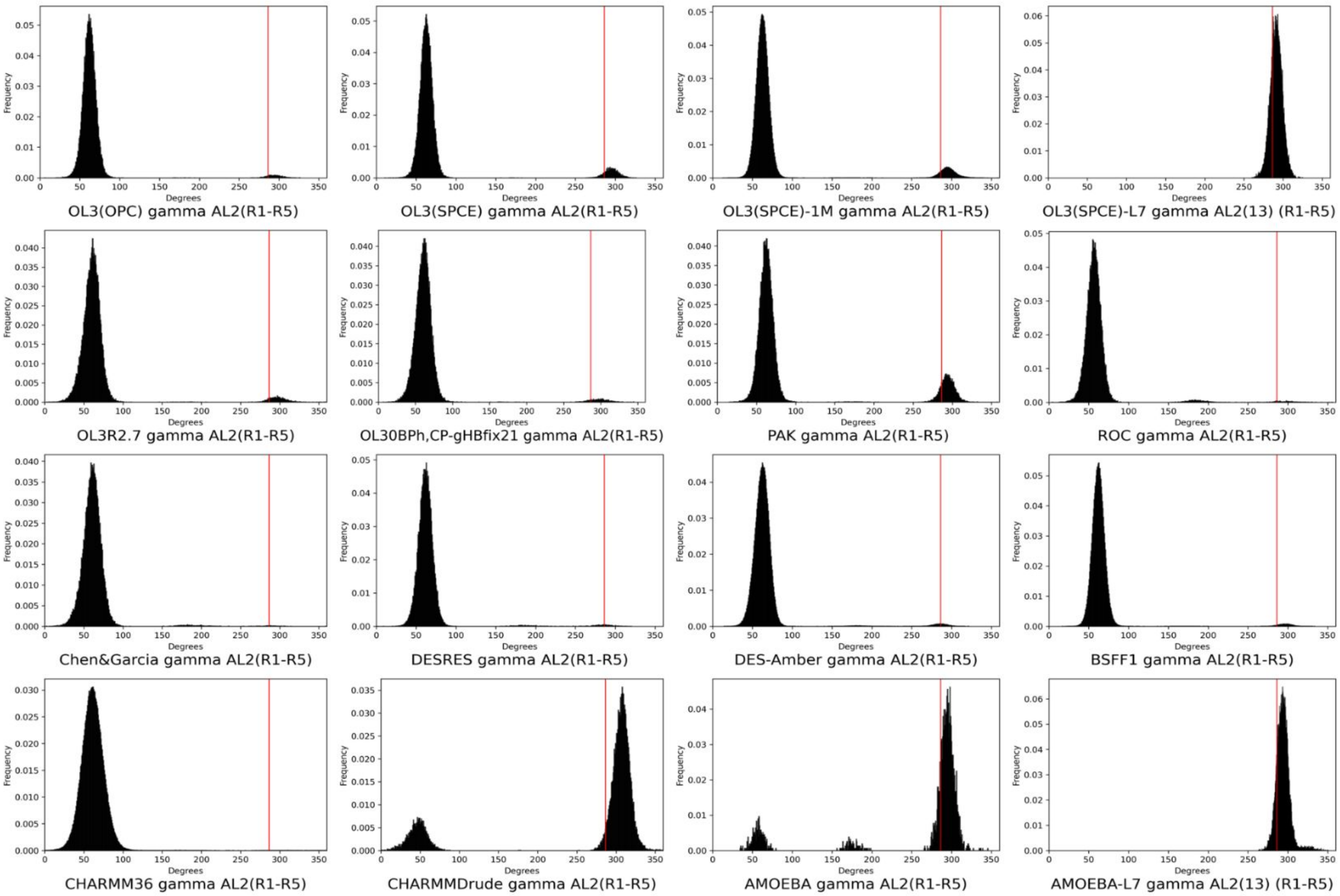

Figure S4B: **Histograms of the backbone dihedral γ of suite G_{L1}/A_{L2} for all tested FFs.** Values for combined simulation ensembles are shown. The vertical red line represents the experimental value (see Table S1).

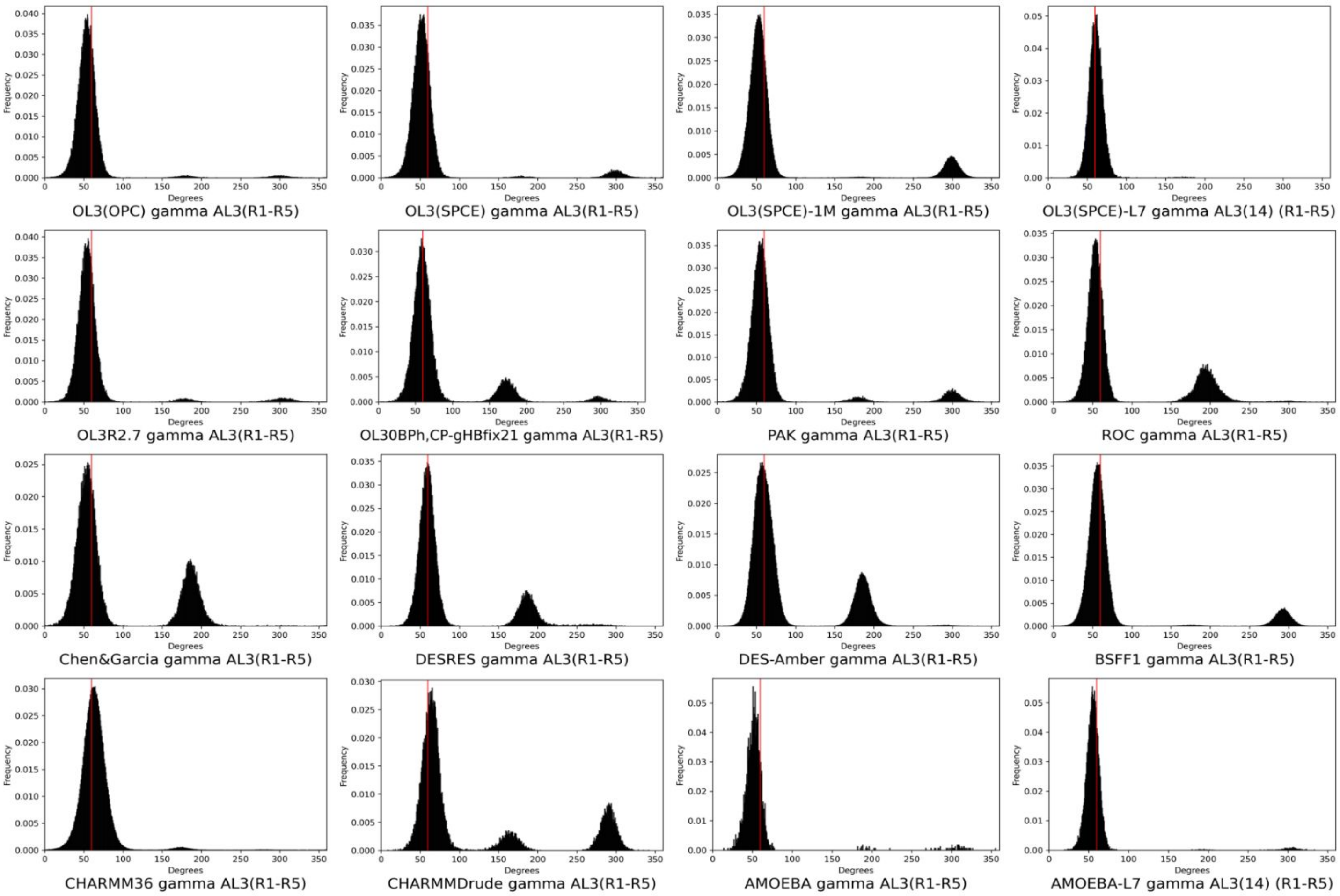

Figure S4C: **Histograms of the backbone dihedral γ of suite A_{L2}/A_{L3} for all tested FFs.** Values for combined simulation ensembles are shown. The vertical red line represents the experimental value (see Table S1)

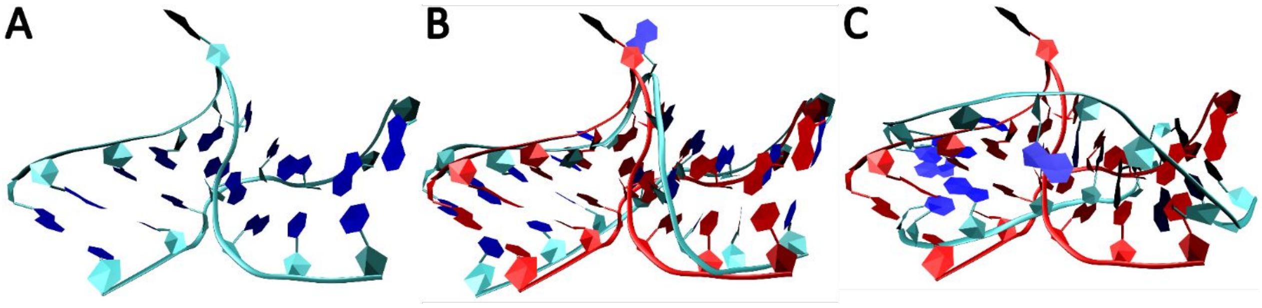

Figure S5: **Example of the Kt-7 unkinking.** (A) The starting structure; (B) Slightly straightened intermediary conformation with the stems still intact (7.37 µs); (C) Unkinked structure with progressive deterioration of the stems (9.68 µs). All simulation figures are taken from the replicate 4 of the DESRES FF simulations. For panels B and C, the starting structure is also shown as a red overlay.

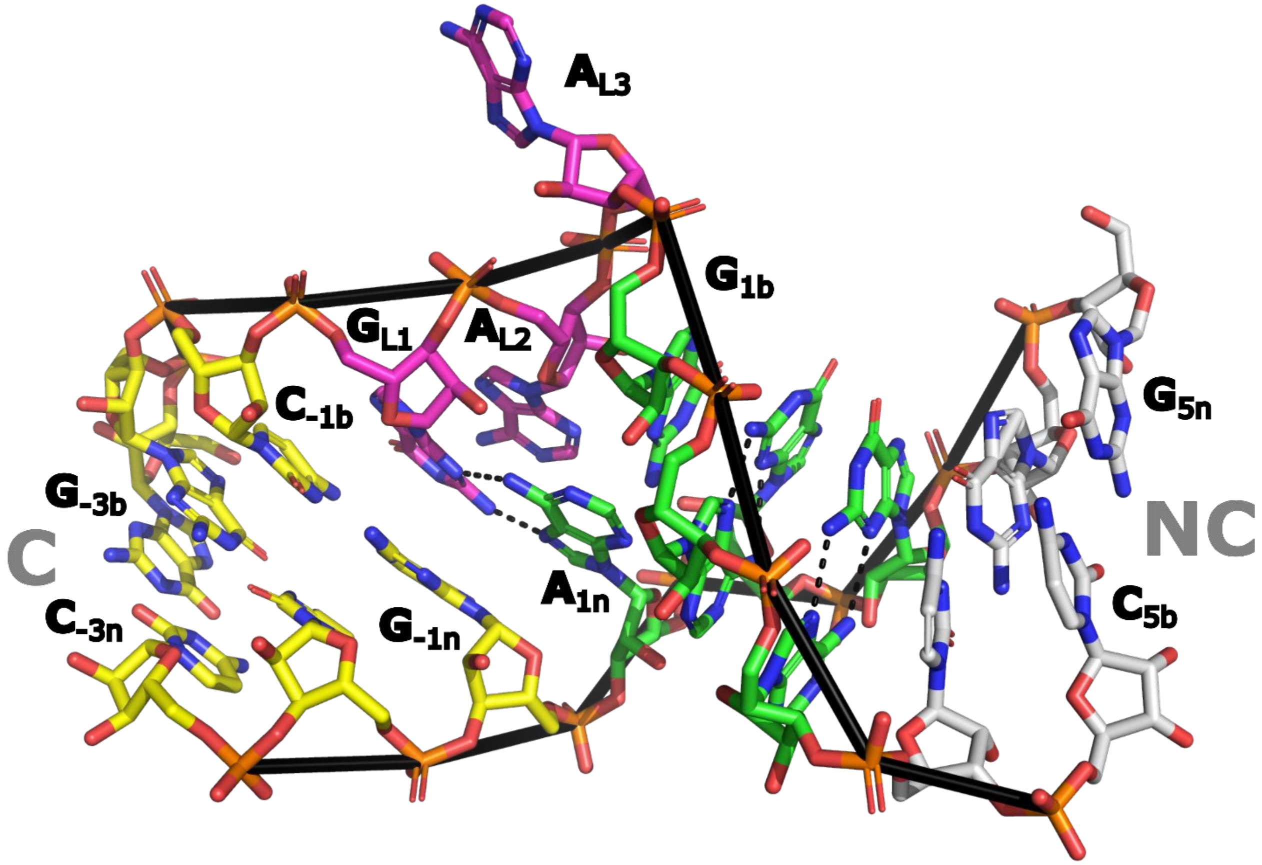

Figure S6: **Formation of a spurious, non-native AG base pair between A_{1n} and G_{L1}.** In several FFs ($OL3_{0BPh, CP}$-gHBfix21, DESRES, and DES-Amber), the native G_{1b}–A_{1n} base pair breaks during unkinking, subsequently leading to formation of a spurious AG base pair between A_{1n} and G_{L1}. The snapshot shown was taken from the DESRES simulation, replicate 1 (1.26 µs), with residues labeled as in Figure 1 (the main text) and the AG base pair H-bonds shown with dashed black lines.

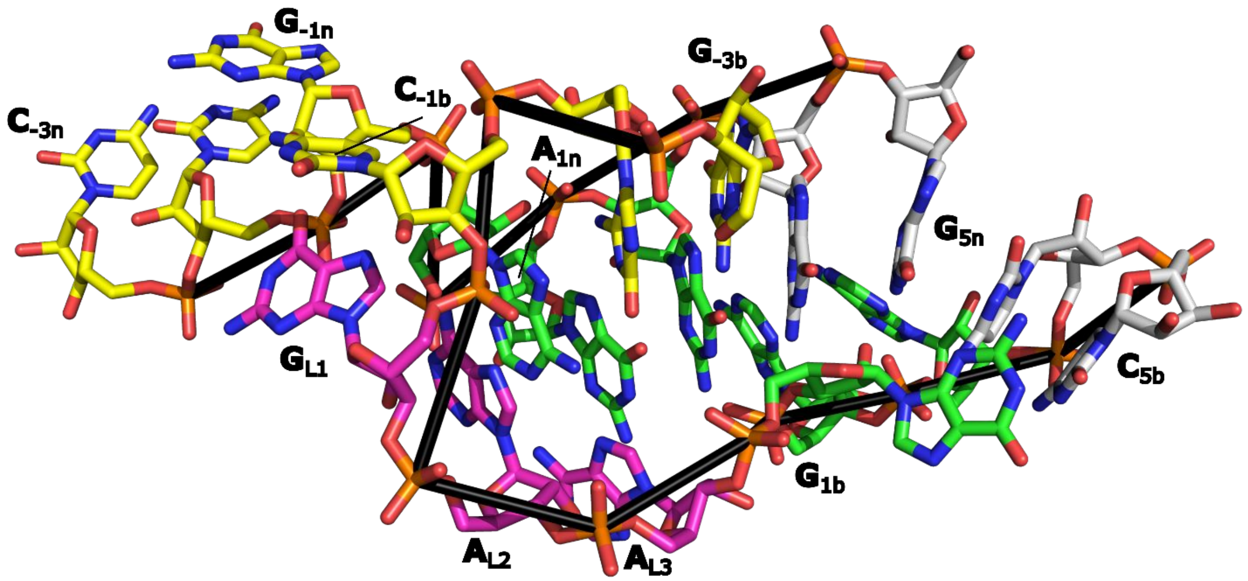

Figure S7: **Severely disrupted Kt-7 structure observed in CHARMM36 simulations.** Virtually all the native H-bonds of both stems are all lost. The structure shown is from replicate 4 (3.03 μs).

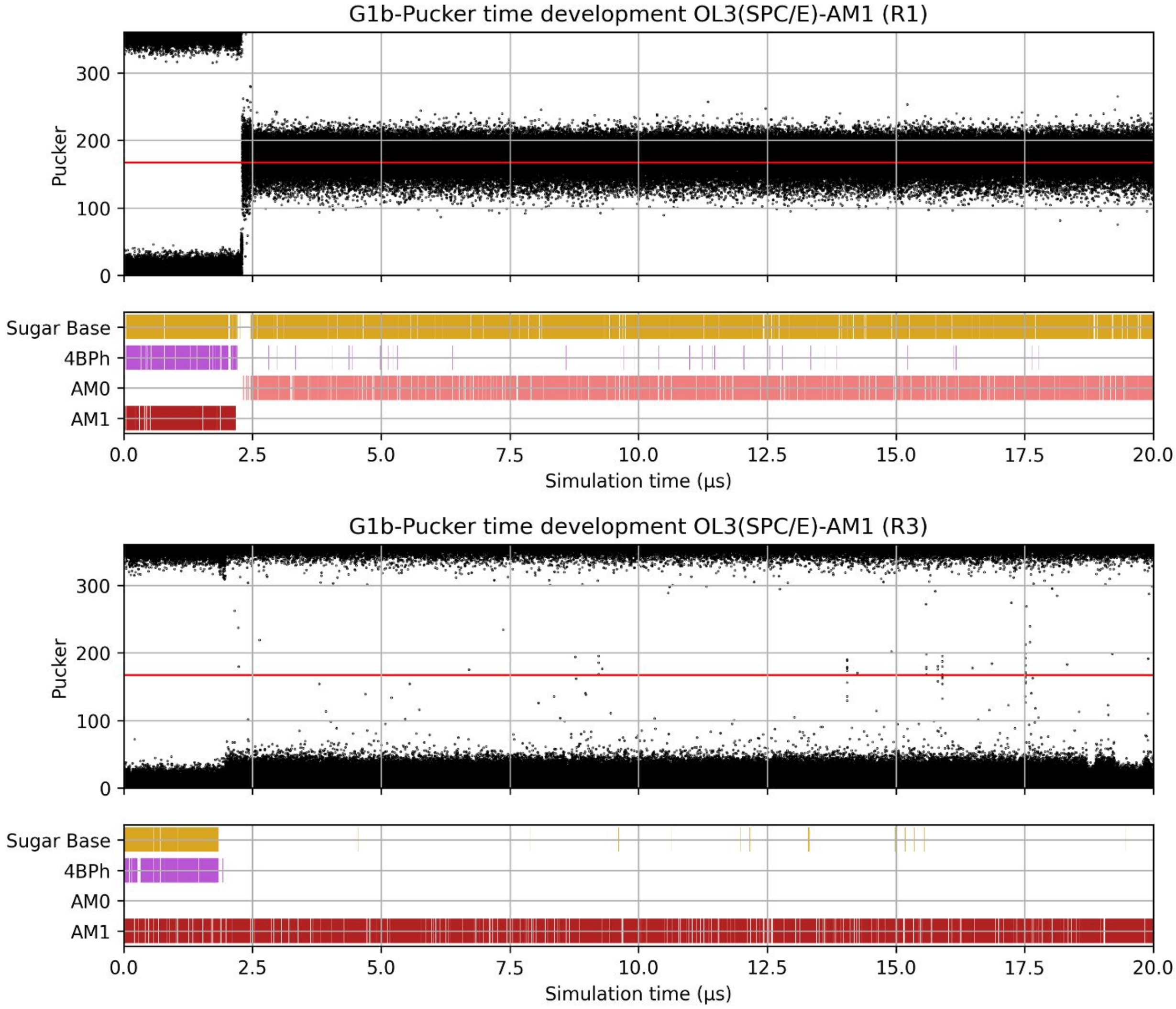

Figure S8: **Time development of the G_{2n} sugar pucker in replicate 1 and 3 of the of OL3-(SPC/E)-AM1 simulations.** The upper plot shows the pucker at each frame with the horizontal red line representing the experimental value (see Table S1). The bottom bar plot depicts presence of the sugar base (G_{1b}(O2')-G_{2n}(N2) and the 4BPh interaction (A_{2b}(OP2)-G_{3n}(N1/N2)) using yellow and purple color, respectively. Formation of A minor 0 or A minor I interaction is then shown in lighter and darker red, respectively. Changes in G_{2n} sugar puckers are highly correlated with changes among these interactions. The other replicates show identical correlations.

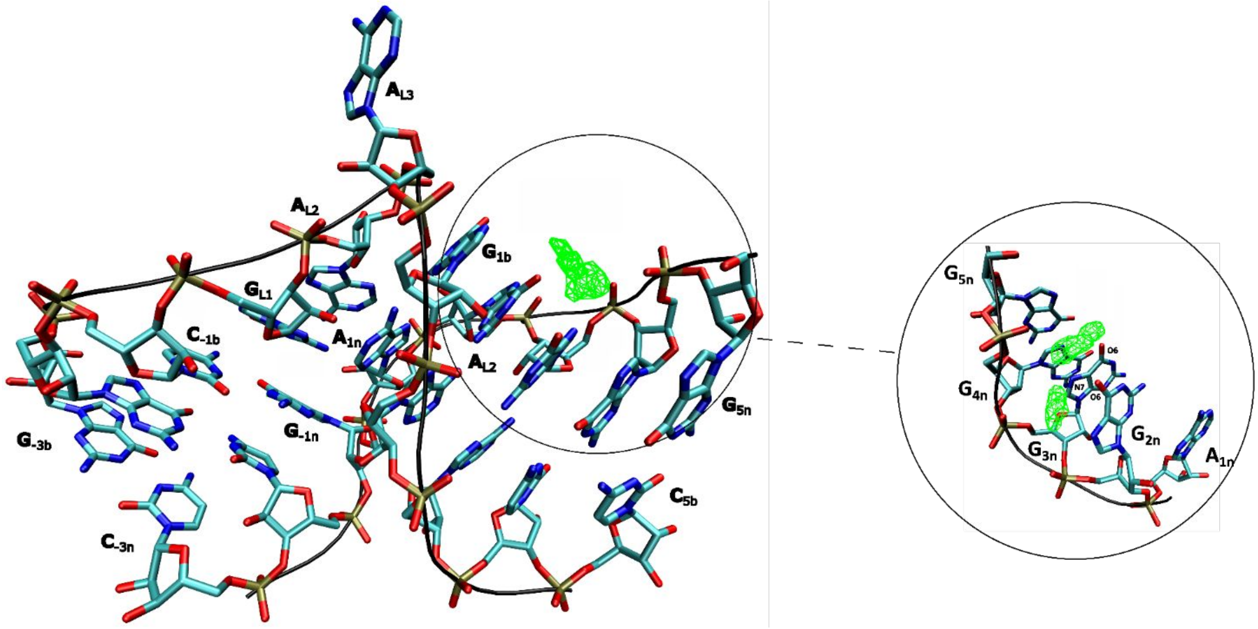

Figure S9: **Visualization of the most densely occupied cation binding sites in MD simulations of Kt-7**. The density map of K^+ ions (shown as green wireframe) reveals the ion-binding sites near atoms G_{2n}(O6), G_{3n}(O6) and G_{3n}(N7) (shown in the inset). Carbon atoms are colored in cyan. The shown density map was generated based on one of the OL3(OPC) simulations, however, similar positioning of the ion binding sites was observed with all FFs albeit their exact populations could differ.

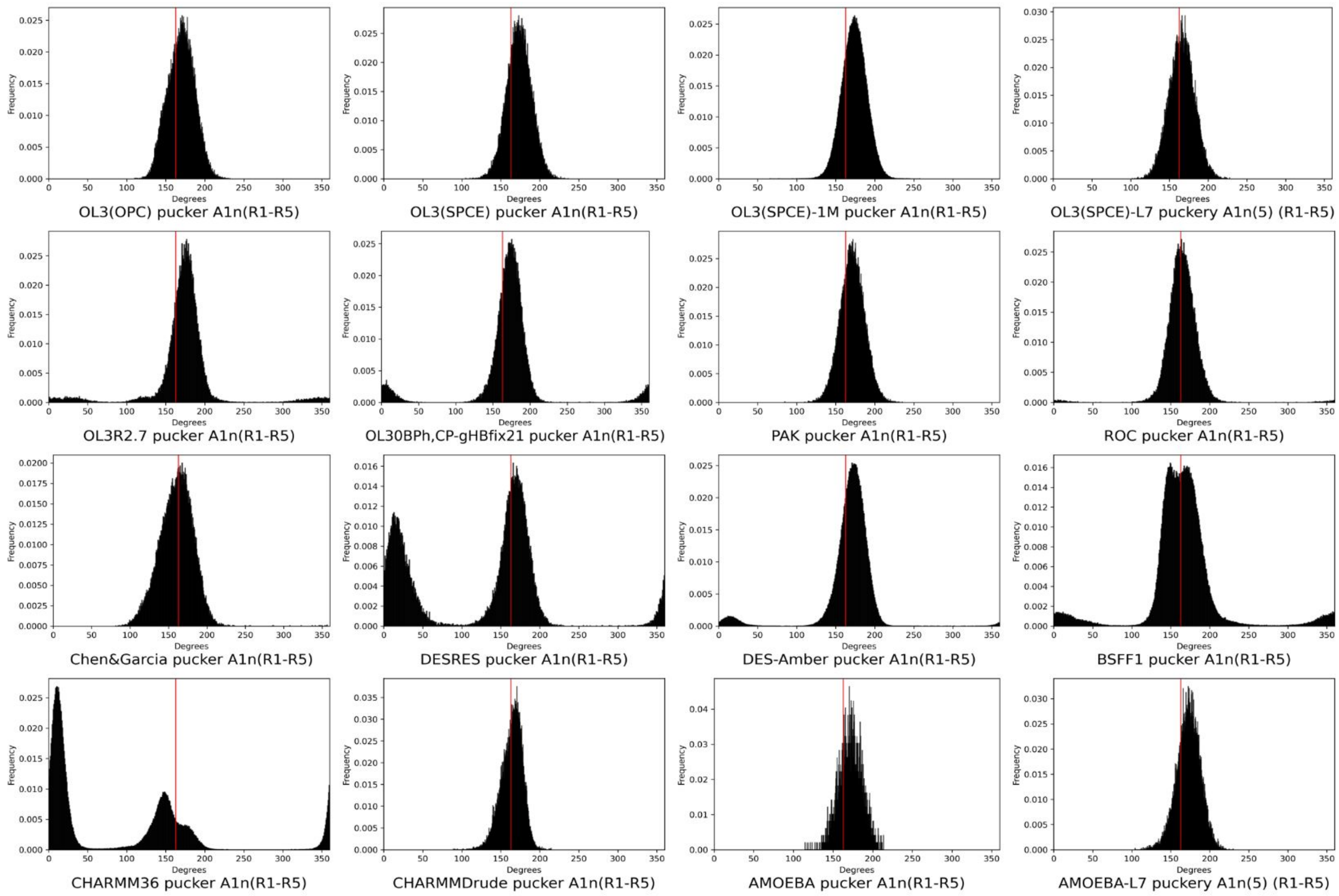

Figure S10A: **Histograms of the pucker of A_{1n} for all tested FFs.** Values for combined simulation ensembles are shown. The vertical red line represents the experimental value (see Table S1).

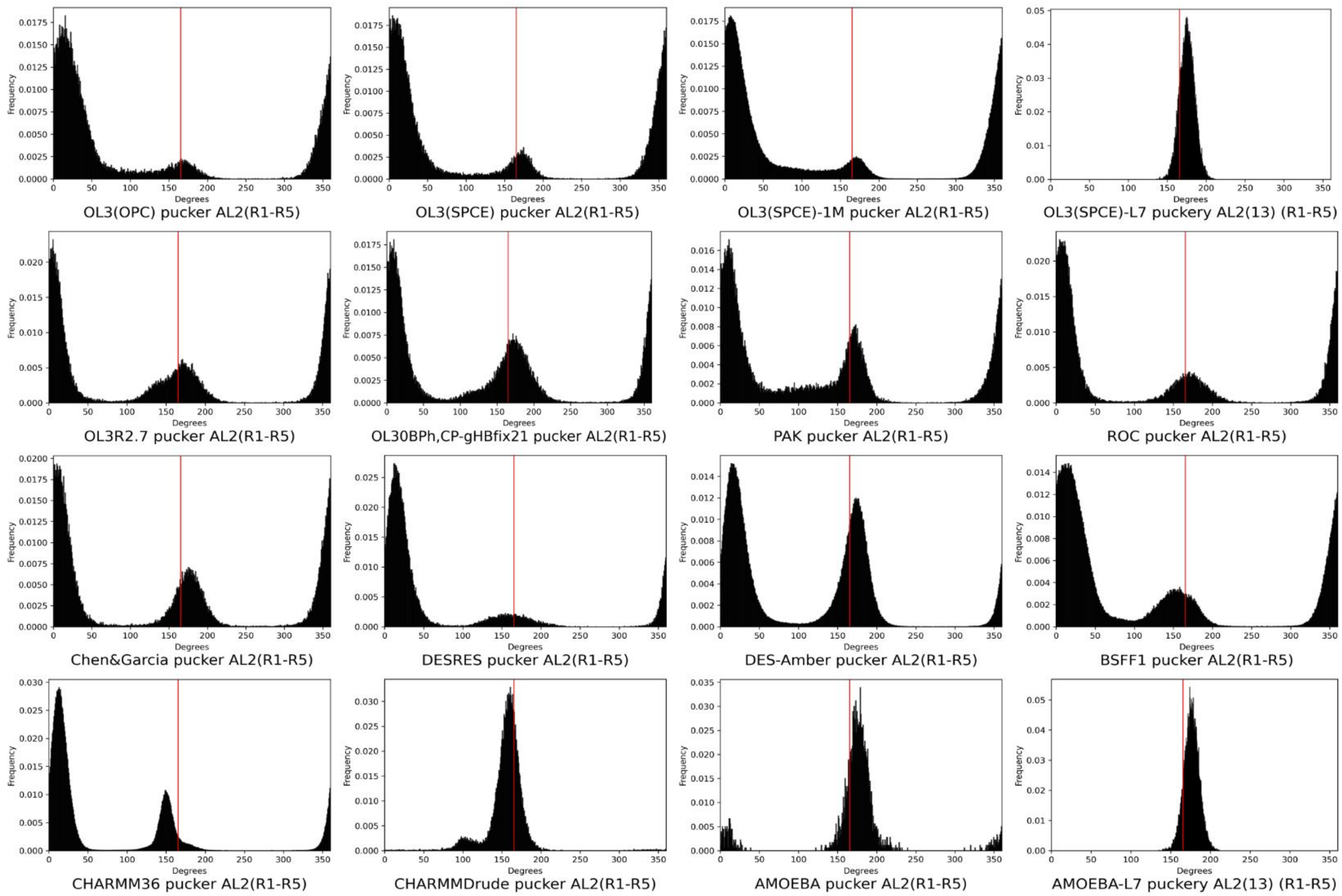

Figure S10B: **Histograms of the pucker of A_{L2} for all tested FFs.** Values for combined simulation ensembles are shown. The vertical red line represents the experimental value (see Table S1).

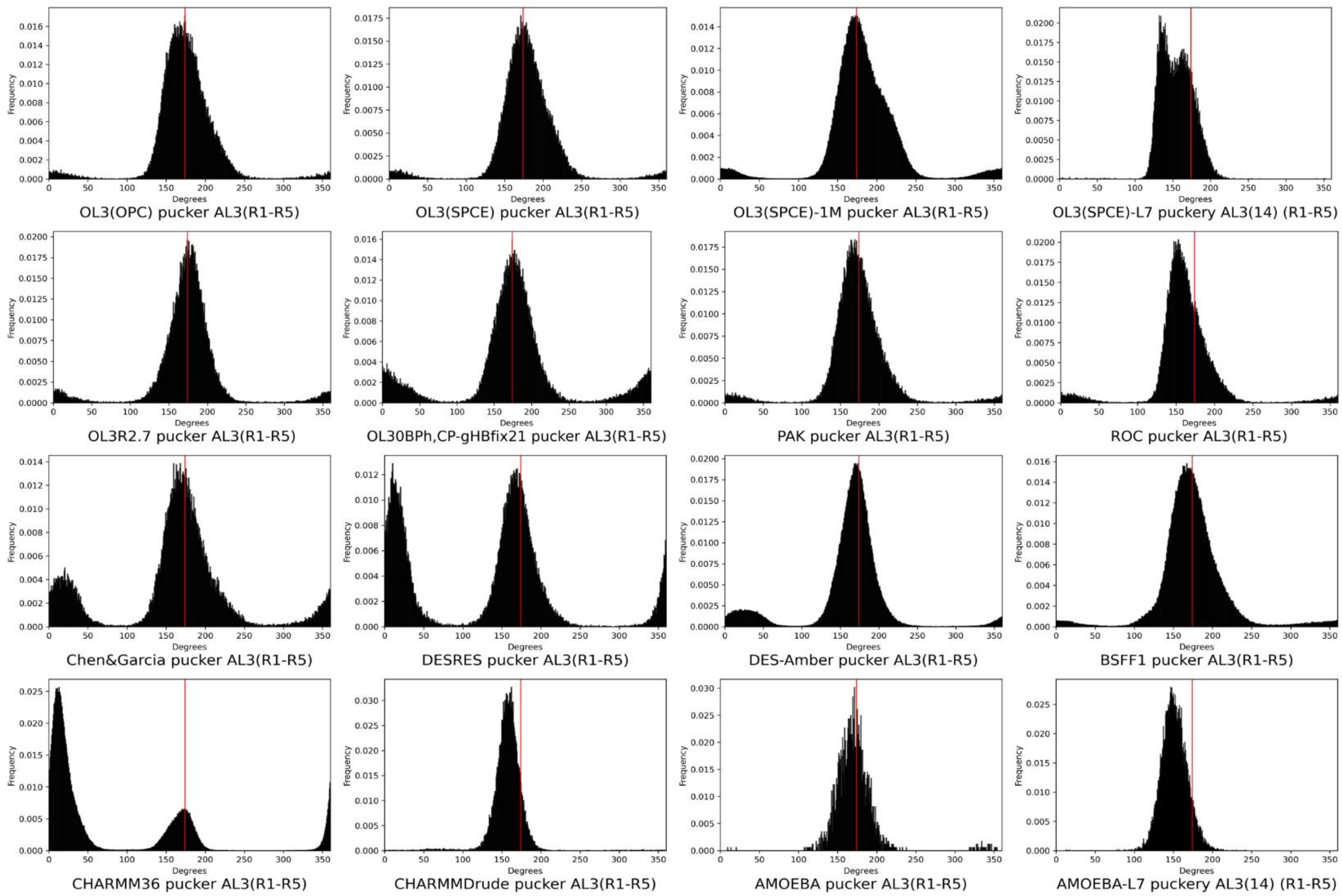

Figure S10C: **Histograms of the pucker of A_{L3} for all tested FFs.** Values for combined simulation ensembles are shown. The vertical red line represents the experimental value (see Table S1).

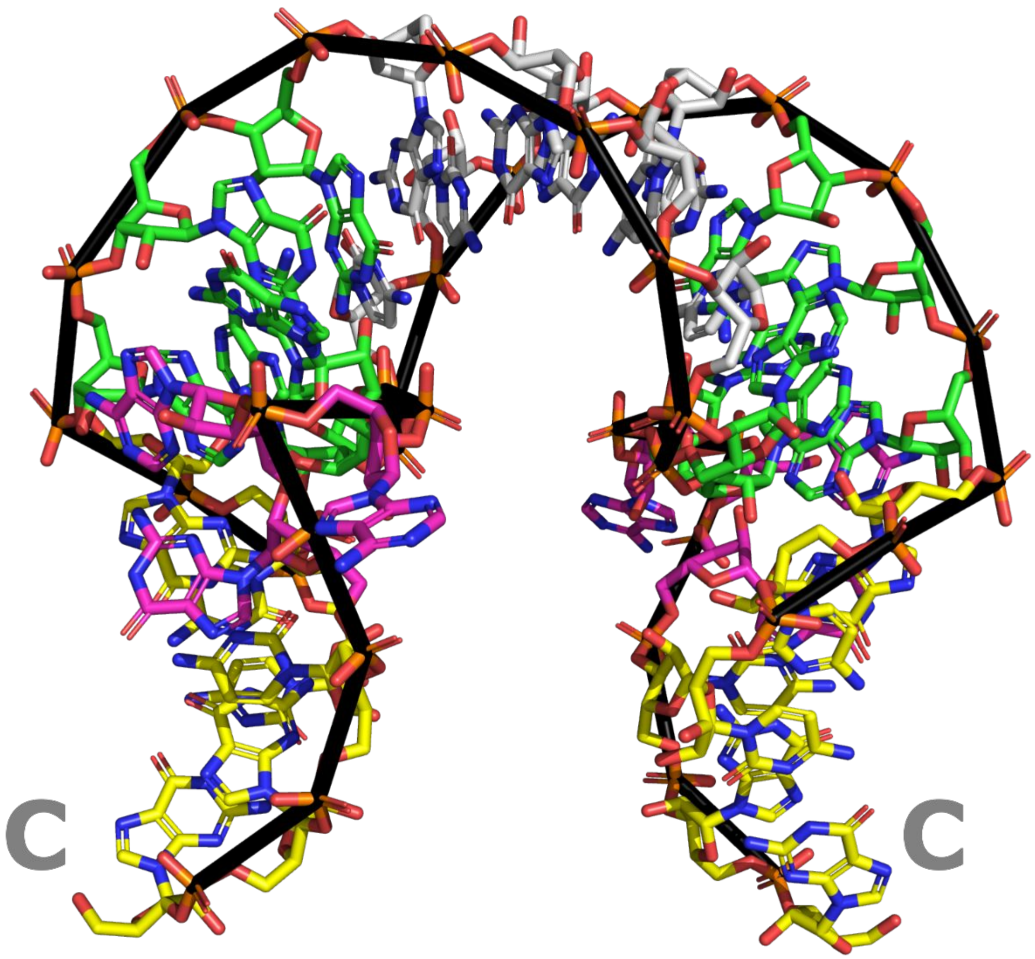

Figure S11: **Non-truncated Kt-7 structure.** When expanded to the biological unit via symmetry operations, the structure deposited as PDB: 4C40 consists of two identical Kt-7 motifs joined through their non-canonical stems. The white residues referring to the Watson-Crick base pairs of the canonical stem depict this interface. The rest of the residues are colored as in Figure 1 in the main text. Each C corresponds to the canonical stem of the joint kink-turns.

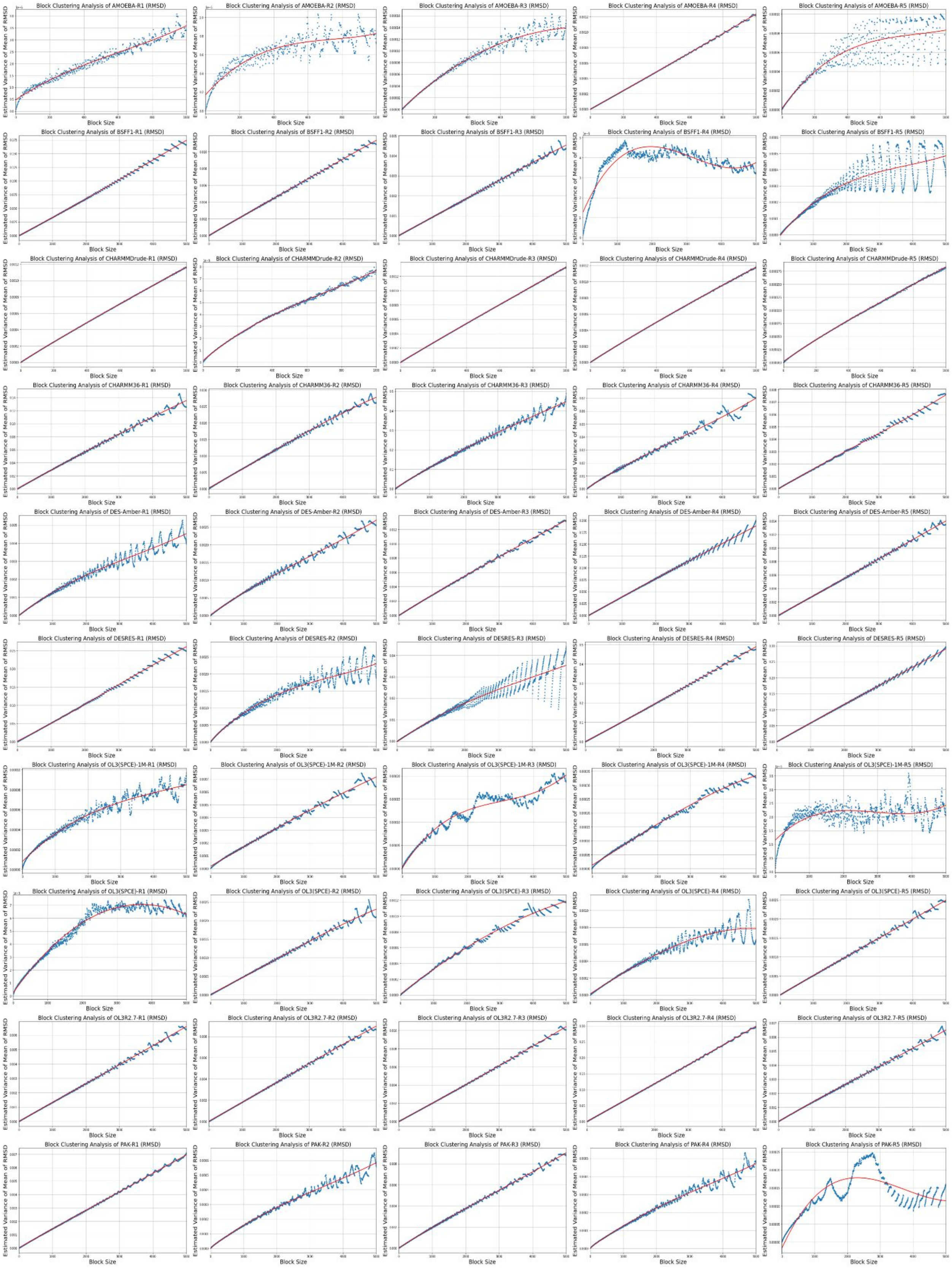
Block Clustering Analysis of AMOEBA-R1 (RMSD)
Block Clustering Analysis of AMOEBA-R2 (RMSD)
Block Clustering Analysis of AMOEBA-R3 (RMSD)
Block Clustering Analysis of AMOEBA-R4 (RMSD)
Block Clustering Analysis of AMOEBA-R5 (RMSD)
Block Clustering Analysis of BSFF1-R1 (RMSD)
Block Clustering Analysis of BSFF1-R2 (RMSD)
Block Clustering Analysis of BSFF1-R3 (RMSD)
Block Clustering Analysis of BSFF1-R4 (RMSD)
Block Clustering Analysis of BSFF1-R5 (RMSD)
Block Clustering Analysis of CHARMMDrude-R1 (RMSD)
Block Clustering Analysis of CHARMMDrude-R2 (RMSD)
Block Clustering Analysis of CHARMMDrude-R3 (RMSD)
Block Clustering Analysis of CHARMMDrude-R4 (RMSD)
Block Clustering Analysis of CHARMMDrude-R5 (RMSD)
Block Clustering Analysis of CHARMM36-R1 (RMSD)
Block Clustering Analysis of CHARMM36-R2 (RMSD)
Block Clustering Analysis of CHARMM36-R3 (RMSD)
Block Clustering Analysis of CHARMM36-R4 (RMSD)
Block Clustering Analysis of CHARMM36-R5 (RMSD)
Block Clustering Analysis of DES-Amber-R1 (RMSD)
Block Clustering Analysis of DES-Amber-R2 (RMSD)
Block Clustering Analysis of DES-Amber-R3 (RMSD)
Block Clustering Analysis of DES-Amber-R4 (RMSD)
Block Clustering Analysis of DES-Amber-R5 (RMSD)
Block Clustering Analysis of DESRES-R1 (RMSD)
Block Clustering Analysis of DESRES-R2 (RMSD)
Block Clustering Analysis of DESRES-R3 (RMSD)
Block Clustering Analysis of DESRES-R4 (RMSD)
Block Clustering Analysis of DESRES-R5 (RMSD)
Block Clustering Analysis of OL3(SPCE)-1M-R1 (RMSD)
Block Clustering Analysis of OL3(SPCE)-1M-R2 (RMSD)
Block Clustering Analysis of OL3(SPCE)-1M-R3 (RMSD)
Block Clustering Analysis of OL3(SPCE)-1M-R4 (RMSD)
Block Clustering Analysis of OL3(SPCE)-1M-R5 (RMSD)
Block Clustering Analysis of OL3(SPCE)-R1 (RMSD)
Block Clustering Analysis of OL3(SPCE)-R2 (RMSD)
Block Clustering Analysis of OL3(SPCE)-R3 (RMSD)
Block Clustering Analysis of OL3(SPCE)-R4 (RMSD)
Block Clustering Analysis of OL3(SPCE)-R5 (RMSD)
Block Clustering Analysis of OL3R2.7-R1 (RMSD)
Block Clustering Analysis of OL3R2.7-R2 (RMSD)
Block Clustering Analysis of OL3R2.7-R3 (RMSD)
Block Clustering Analysis of OL3R2.7-R4 (RMSD)
Block Clustering Analysis of OL3R2.7-R5 (RMSD)
Block Clustering Analysis of PAK-R1 (RMSD)
Block Clustering Analysis of PAK-R2 (RMSD)
Block Clustering Analysis of PAK-R3 (RMSD)
Block Clustering Analysis of PAK-R4 (RMSD)
Block Clustering Analysis of PAK-R5 (RMSD)
Estimated Variance of Mean of RMSD
Block Size

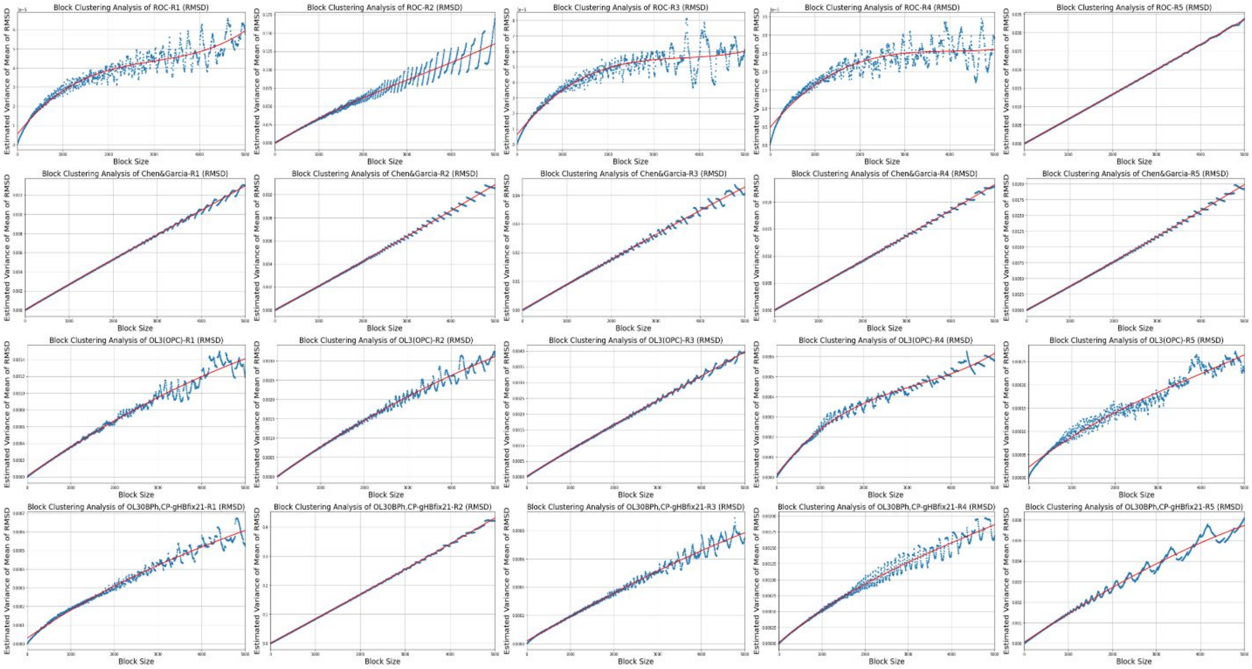

Figure S12: **Block averaging analysis for each replicate of all tested FFs**. Blue graph represents the variance calculates for each block size. The red line represents an interpolation with smoothing factor 0.5 to improve readability.

Supporting Information References

(1) Falb, M.; Amata, I.; Gabel, F.; Simon, B.; Carlomagno, T. Structure of the K-turn U4 RNA: a combined NMR and SANS study. *Nucleic Acids Res.* **2010**, *38* (18), 6274-6285. DOI: 10.1093/nar/gkq380 (acccessed 3/31/2025).

(2) Huang, L.; Lilley, D. M. The molecular recognition of kink-turn structure by the L7Ae class of proteins. *RNA* **2013**, *19* (12), 1703-1710. DOI: 10.1261/rna.041517.113.

(3) Frohlking, T.; Mlýnský, V. c.; Janeček, M.; Kührová, P.; Krepl, M.; Banáš, P.; Šponer, J. í.; Bussi, G. Automatic learning of hydrogen-bond fixes in the AMBER RNA force field. *J. Chem. Theory Comput.* **2022**, *18* (7), 4490-4502. DOI: 10.1021/acs.jctc.2c00200.

(4) Kuhrova, P.; Mlynsky, V.; Zgarbová, M.; Krepl, M.; Bussi, G.; Best, R. B.; Otyepka, M.; Sponer, J.; Banas, P. Improving the performance of the amber RNA force field by tuning the hydrogen-bonding interactions. *J. Chem. Theory Comput.* **2019**, *15* (5), 3288-3305. DOI: https://doi.org/10.1021/acs.jctc.8b00955.

(5) Huang, L.; Lilley, D. M. The kink turn, a key architectural element in RNA structure. *J. Mol. Biol.* **2016**, *428* (5), 790-801. DOI: https://doi.org/10.1016/j.jmb.2015.09.026.

(6) Zgarbová, M.; Šponer, J.; Jurečka, P. Z-DNA as a Touchstone for Additive Empirical Force Fields and a Refinement of the Alpha/Gamma DNA Torsions for AMBER. *J. Chem. Theory Comput.* **2021**, *17* (10), 6292-6301. DOI: 10.1021/acs.jctc.1c00697.

(7) Altona, C.; Sundaralingam, M. Conformational analysis of the sugar ring in nucleosides and nucleotides. New description using the concept of pseudorotation. *J. Am. Chem. Soc.* **1972**, *94* (23), 8205-8212. DOI: 10.1021/ja00778a043.
